# Dietary intake of enrofloxacin promotes the spread of antibiotic resistance from food to simulated human gut

**DOI:** 10.1093/ismejo/wraf045

**Published:** 2025-03-23

**Authors:** Qing Wang, Changzhen Liu, Yan Sun, Xuli Li, Weimin Gu, Na Wang, Shaojing Sun, Yi Luo

**Affiliations:** College of Energy and Environmental Engineering, Hebei Key Laboratory of Air Pollution Cause and Impact, Hebei Engineering Research Center of Sewage Treatment and Resource Utilization, Hebei University of Engineering, Handan 056038, China; College of Energy and Environmental Engineering, Hebei Key Laboratory of Air Pollution Cause and Impact, Hebei Engineering Research Center of Sewage Treatment and Resource Utilization, Hebei University of Engineering, Handan 056038, China; College of Energy and Environmental Engineering, Hebei Key Laboratory of Air Pollution Cause and Impact, Hebei Engineering Research Center of Sewage Treatment and Resource Utilization, Hebei University of Engineering, Handan 056038, China; College of Energy and Environmental Engineering, Hebei Key Laboratory of Air Pollution Cause and Impact, Hebei Engineering Research Center of Sewage Treatment and Resource Utilization, Hebei University of Engineering, Handan 056038, China; College of Energy and Environmental Engineering, Hebei Key Laboratory of Air Pollution Cause and Impact, Hebei Engineering Research Center of Sewage Treatment and Resource Utilization, Hebei University of Engineering, Handan 056038, China; Key Laboratory of Pesticide Environmental Assessment and Pollution Control, Nanjing Institute of Environmental Science, Ministry of Ecology and Environment of the People's Republic of China, Nanjing 210042, China; College of Energy and Environmental Engineering, Hebei Key Laboratory of Air Pollution Cause and Impact, Hebei Engineering Research Center of Sewage Treatment and Resource Utilization, Hebei University of Engineering, Handan 056038, China; State Key Laboratory of Water Pollution Control and Green Resource Recycling, School of the Environment, Nanjing University, Nanjing 210093, China

**Keywords:** antibiotic-resistant bacteria, antibiotic resistance genes, colonization resistance, dietary exposure, enrofloxacin, horizontal gene transfer, simulated human intestine

## Abstract

Antibiotic residues are commonly found in food. The effect of dietary exposure to veterinary antibiotics on the transmission of antibiotic-resistant bacteria and antibiotic resistance genes (ARGs) from food to humans is unknown. We found that dietary exposure to enrofloxacin reduced microbial diversity, interactions, and the immune responses; weakened the colonization resistance of the resident microbiota; and promoted the colonization of exogenous *Escherichia coli* K-12 MG1655 in the simulated human intestine both *in vitro* and *in vivo* experiments in mice. In addition to the growth advantages for potential most likely bacterial hosts of ARGs under enrofloxacin exposure, the dietary exposure to enrofloxacin promoted horizontal transfer of resistance plasmids and altered the simulated human gut antibiotic resistome in a time-dependent manner. Collectively, these findings demonstrated that dietary intake of enrofloxacin promoted the colonization of *E. coli* K-12 MG1655 in the simulated human intestine and the horizontal transfer of ARGs, highlighting the risk of antibiotic resistance transmission from food to humans mediated by dietary exposure to veterinary antibiotics.

## Graphical Abstract

**Graphical Abstract:**
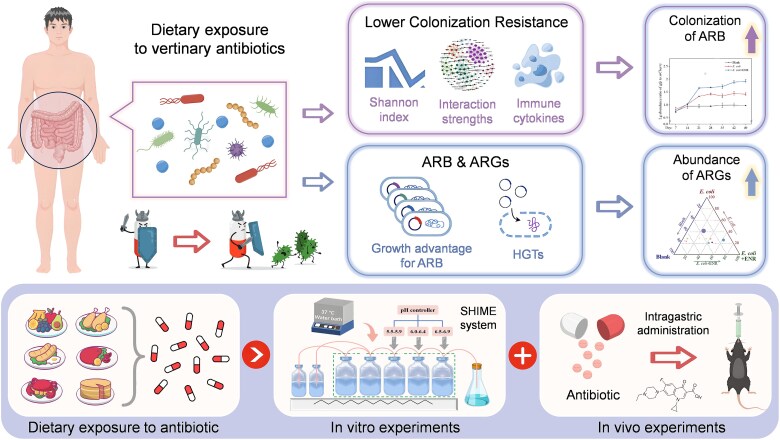

## Introduction

Antimicrobial resistance has long been confirmed to be a global health crisis [1, 2]. In recent years, the incidence of foodborne diseases has been reduced by implementing food safety measures from farm to fork. Antibiotics are widely used in animal husbandry to treat and prevent diseases, as well as to promote growth or improve production performance. The misuse and abuse of antibiotics might lead to the presence of veterinary antibiotic residues in edible tissues [3–5], which can induce changes in the intestinal microbiota in the diet or upon exposure to antibiotics [6, 7]. The residual concentrations of enrofloxacin in aquatic products and raw milk are 148.4 ng/g and 161.2 ng/ml [8, 9], respectively, which exceed 100 ng/g (the maximum residue limit recommended by the European Commission for food) [10]. The daily intake of enrofloxacin by the general population through diet and drinking water ranges from 8.39 to 698 μg/day [11, 12].

In addition to the antibiotic residues in food, human consumption of food contaminated with bacteria and viruses can disturb the structure of the flora and cause foodborne diseases [13]. Many types of foodborne pathogenic bacteria, including *Salmonella*, *Clostridium botulinum*, and *Escherichia coli*, pose considerable threats to human health and safety [14]. Food and food products may be carriers of antibiotic-resistant bacteria (ARB) and antibiotic resistance genes (ARGs), posing a potential risk to human health [15, 16]. *Mcr-1*-positive *Salmonella* binds to gavage recipient bacteria and mouse intestinal flora to transfer *mcr-1* during food consumption [17]. Thus, the emergence of a large number of ARBs in food has made foodborne acquired antibiotic resistance the focus of worldwide attention.

Various studies have shown that the use of antibiotics is associated with the emergence of ARBs in animals and their transmission to humans, especially through the food chain [18, 19]. Although antibiotics are undoubtedly degraded by human digestive enzymes, the antibiotics that enter the intestine through the digestive system also stress the gut microbiota [20]. In our previous study, four veterinary antibiotics—enrofloxacin, chlortetracycline, sulfachlorpyridazine, and sulfadimethoxine—were isolated from the intestines of healthy people, indicating that they are ingested through animal-derived foods [21, 22]. Moreover, vancomycin exposure increases the number of pathways associated with human diseases in the gut microbiota and reduces the abundance of ARGs [23]. The evolutionary adaptation of plasmid-carrying bacteria to antibiotic (ampicillin and kanamycin) exposure promotes their colonization in the mouse intestine and subsequent plasmid transfer between intestinal bacteria [24]. However, the extent to which low-concentration, long-term exposure to veterinary antibiotics through the food chain (under dietary exposure) affects the production and spread of ARBs in the human gut has not yet been agreed upon.

In this work, we addressed the influence of dietary exposure to veterinary antibiotic residues in food on the antibiotic resistance of simulated human gut microbiota with *in vitro* experiments in a simulator of the human intestinal microbial ecosystem (SHIME) and *in vivo* experiments in mice. In addition, the effects of exposure time to veterinary antibiotics in food on the intestinal microbiota and antibiotic resistome were investigated. The results obtained in this study provided insights into the colonization of *E. coli* and horizontal transfer of ARGs under dietary exposure to veterinary antibiotics, highlighting the risk of antibiotic resistance transmission from food to humans mediated by dietary exposure to veterinary antibiotics.

## Materials and methods

### Antibiotic, strains, and culture conditions

Enrofloxacin, one of the fluoroquinolone antibiotics, has moderate and variable effects on various bacteria by binding to the bacterial DNA gyrase subunit A to inhibit the activity of the enzyme and affect the processes of DNA melting, cutting, and resealing [25]. The addition of 70 μg/day of enrofloxacin for 14 days to the SHIME system corresponded to the daily dietary exposure levels observed in humans in China, as reported in previous studies [11, 12]. However, a limitation of this study was that the SHIME system could only simulate half the volume of an adult human gut [26, 27]. Based on the equivalent dose coefficient method in ecotoxicology and pharmacology, the enrofloxacin exposure dose used in mouse experiments was 0.8 mg/day.

The multidrug-resistant *E. coli* K-12 MG1655 (referred to as *E. coli* in this study) strain carried the broad-host-range conjugative plasmid RP4. The chromosome of this strain was labeled with the *mCherry* and *lacI^q^* genes. The RP4 plasmid contained resistance genes for ampicillin, kanamycin, and tetracycline, along with a *gfp* gene under the regulation of a promoter repressed by the chromosomally encoded *lacI^q^*. Consequently, due to the specific expression of the *mCherry* gene and the repression of *gfp* expression, *E. coli* MG1655 exhibited only red fluorescence. Therefore, the abundance and intensity of green fluorescence served as indicators of horizontal plasmid transfer. Based on our previous studies [13, 28] and other published articles [29, 30], this approach has been widely used to track plasmid transfer.

*Escherichia coli* was cultured overnight at 37°C in LB medium supplemented with 100 μg/ml ampicillin, 60 μg/ml kanamycin, and 20 μg/ml tetracycline [13]. The bacterial mixture was subsequently centrifuged at 10 000 rpm for 5 min, after which the supernatant was discarded. The residues were washed with phosphate buffer solution (PBS). The above operation was repeated twice. Finally, the obtained cell precipitate was resuspended in sterile PBS buffer with an OD_600_ value of 1.0 for subsequent use.

### *In vitro* simulator of the human intestinal microbial ecosystem model experiments

**Figure 1:**
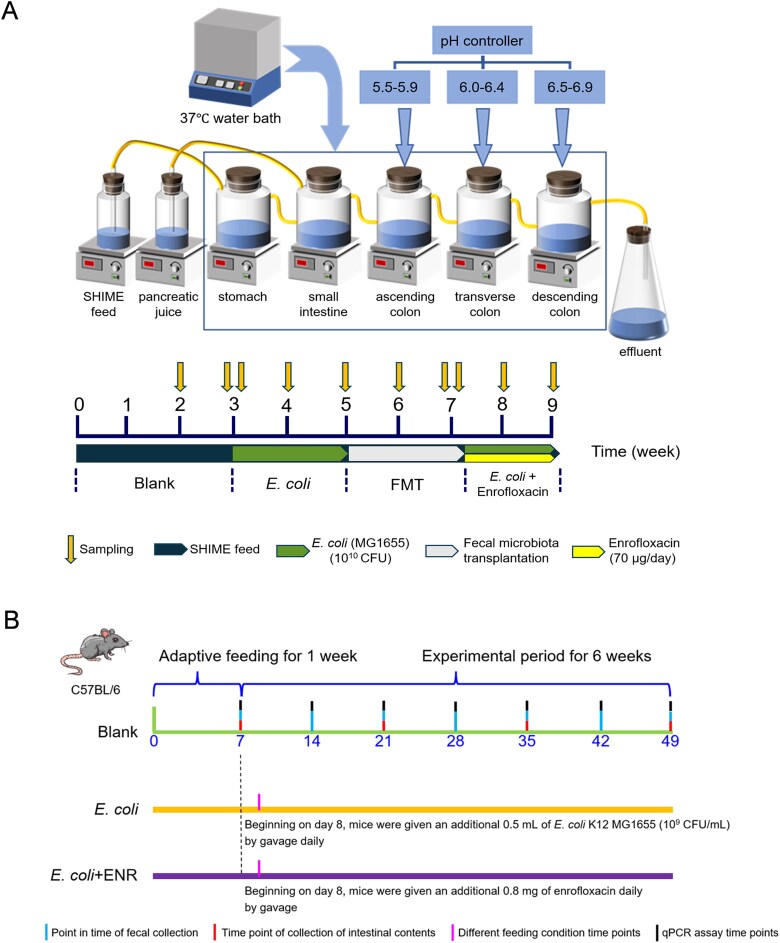
(A and B) Schematic of *in vitro* and *in vivo* experiments. SHIME system simulator comprises five glass reactors representing stomach, small intestine, ascending colon, transverse colon, and descending colon. Temperature is maintained at 37°C using a thermostatic circulator, and gastrointestinal peristalsis is simulated with magnetic stirrers (A). Experimental design schematic for *in vivo* mice experiments (B).

The SHIME simulator was used in this study according to previous studies [31, 32]. The compositions of the SHIME culture and pancreatic juice are listed in Supplementary Tables S1 and S2, respectively. In this study, the experimental cycle included 9 weeks. A total of four periods, namely, the Blank, *E. coli*, fecal microbiota transplantation (FMT), and *E. coli +* enrofloxacin (ENR) periods, were conducted. The specific experimental process is shown in Fig. 1A. More detailed information can be found in Supplementary Text S1.

### *In vivo* mice experiments

 Six-week-old specific pathogen free-grade C57BL/6 female mice (~18–22 g) were purchased from Jiangsu Yinowei Biomedical Research Institute. A total of 36 mice were used in this study. The mice were raised in separate cages and allowed to drink freely. The *in vivo* experiments on mice lasted for a total of 7 weeks. The sampling design process is shown in Fig. 1B. The detailed groups and dosages are listed in Supplementary Table S3. More detailed information can be found in Supplementary Text S2.

### DNA extraction and quantitative PCR amplification analysis

The collected samples were extracted using a fecal DNA mini-extraction kit (HiPure Stool DNA Kit) to extract total DNA. Afterward, the DNA concentration and quality were determined using a Nanophotometer (N60, Implen, Germany). All the DNA samples were stored at −80°C for further analysis. Quantitative PCR amplification (qPCR) was performed using a Bio-Rad iQ5 instrument (Bio-Rad, Hercules, CA). For qPCR, a 20 μl reaction system was used, and the composition of the system was as follows: 10 μl of 2 × SYBR qPCR Master Mix, 0.4 μl of F/R-primer, 8.2 μl of ddH_2_O, and 1 μl of DNA template, with three parallel samples for each sample. Details of primer sequences of the target genes (*gfp*, *mCherry*, and 16S rRNA) and hybridization temperatures are shown in Supplementary Tables S4 and S5, respectively.

### High-throughput sequencing of 16S rRNA genes, metagenomic sequencing, and bioinformatic analysis

The extracted DNA samples were sent to Beijing Baimaike Biotechnology Co., Ltd for metagenome sequencing. The V3-V4 region of the bacterial 16S ribosomal RNA gene was amplified by PCR using an NovaSeq 6000 system (Illumina). The Supplementary Text S3 includes details for this method. Metagenomic sequencing was performed with an Illumina sequencer. The clean reads were assembled, the coding genes were predicted, and the nonredundant gene sets were constructed. The nonredundant gene set results from the *in vitro* SHIME model experiments and *in vivo* mice experiments are listed in Supplementary Tables S6 and S7, respectively. The Nr, KEGG, VFDB, and CARD databases were used for gene function annotation. More bioinformatics analysis is provided in Supplementary Text S4. Identification of ARG potential most likely bacterial hosts is shown in Supplementary Text S5 according to our previous study [33].

### Fluorescence microscope and flow cytometry

The collected mouse feces were physically crushed and ground, and then PBS buffer solution was added to obtain crude extract. The crude extract was centrifuged, filtered, and purified to obtain a suspension of fecal bacteria. A fluorescence microscope (OLYMPUS BX53) was used to observe the red (*E. coli*) and green fluorescent bacteria (bacteria carrying the RP4 antibiotic resistance plasmid through horizontal gene transfer) extracted from mouse feces. To sort the *gfp*-expressing transconjugants by fluorescence activated cell sorting (FACS), a flow cytometer (FACSAri Fusion, BD, USA) was used to separate cells based on green fluorescence signals. The donor strain (*E. coli MG1655*, *mCherry*^+^, *gfp*^−^), which only exhibited red fluorescence, was used to define the Q1 region, ensuring accurate differentiation of transconjugant (Q3, *gfp*^+^) and recipient (Q4, no fluorescence) populations in subsequent analyses (Supplementary Fig. S2). The conjugation transfer rate of plasmids was calculated using the following formula based on the results obtained through flow cytometer: number of conjugates (green fluorescence)/number of bacteria in the gut microbiome (no fluorescence) [34]. Supplementary Texts S6 and S7 include details of these methods.

### Detection of cytokines and enrofloxacin

Four cytokines, namely, IL-6, IL-8, IL-1β, and TNF-α, were measured by enzyme-linked immunosorbent assay according to a previous study [6]. The detailed information is provided in Supplementary Text S8 and Supplementary Table S8. Ultrasonic extraction was employed to isolate residual enrofloxacin from mouse feces. Enrofloxacin was quantified by high-performance liquid chromatography-mass spectrometry (HPLC-MS/MS) (1290-6495, Agilent, USA). The detailed information about the detection of enrofloxacin is provided in Supplementary Text S9 and Supplementary Table S9.

### Statistical analysis

Data were expressed as mean ± standard deviation (SD). SPSS was used to calculate the statistical data. R version 4.2.3 was used to conduct the network analysis. The Code for correlation network analysis is shown in Supplementary Text S10. Origin 2024 and Gephi (V 0.10) were used for visualization analysis. Principal component analysis (PCA) was performed for between-group differential microbiome compositions. One-way analysis of variance (ANOVA) and independent samples *t*-tests were performed for group comparisons, and *P* values were corrected using the Benjamini–Hochberg method [35]. *P* < .05 was considered statistically significant. All experiments were conducted in triplicate.

## Results

### Colonization of *Escherichia coli* and horizontal transfer of resistance plasmid

**Figure 2:**
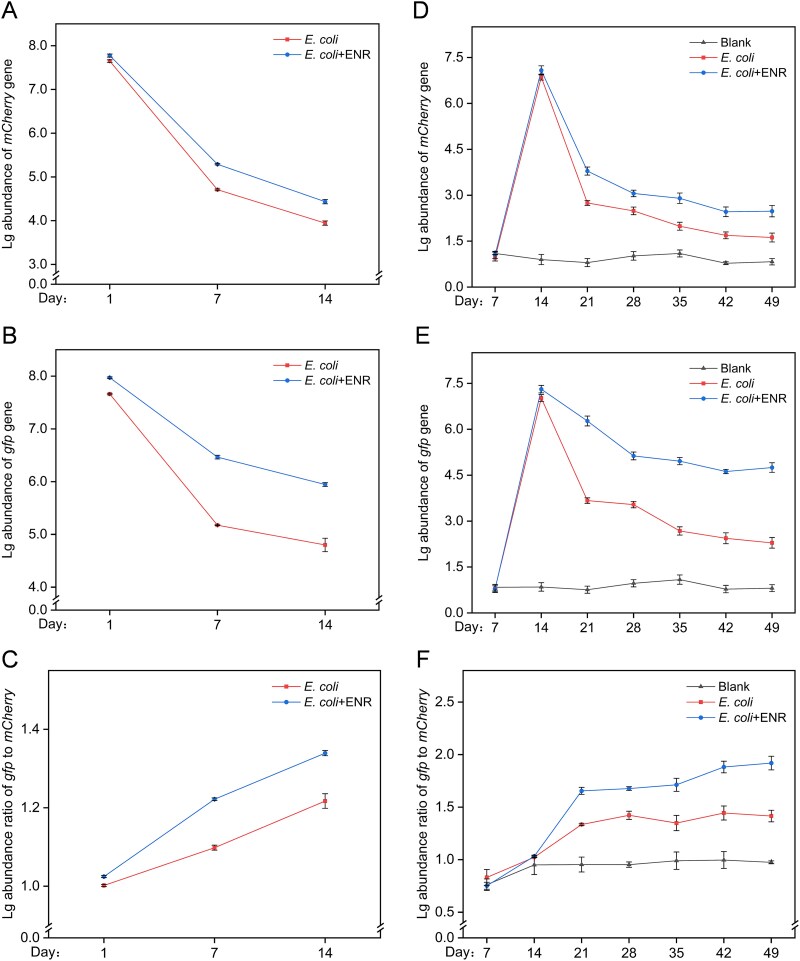
The abundance of *gfp* and *mCherry* (log10 transfer) obtained by qPCR both in SHIME system and mice experiments. The *x*-axis indicates the cumulative duration (in days) of exposure to *E. coli* or coexposure with enrofloxacin. Logarithmic abundance of *mCherry* (A), *gfp* (B), and the logarithmic abundance ratio of *gfp* gene to *mCherry* gene (C) in the ascending colon for the SHIME system. Logarithmic abundance of *mCherry* (D), *gfp* (E), and the logarithmic abundance ratio of *gfp*/*mCherry* (F) in mouse feces. Each data point in the line chart represents the mean ± standard deviation (SD) of three biological replicates.

Quantitative PCR (qPCR) analysis was performed on samples to determine the abundance of *mCherry* and *gfp* both in the SHIME experiments and in mice experiments. In total, a decrease in the abundance of the *mCherry* and *gfp* genes was observed in the initial stage of cultivation because the colonization of *E. coli* in the SHIME system and mouse gut was resisted by the resident bacteria in the gut microbiome. As the cultivation time prolonged, the downward trend gradually slowed down and tended to stabilize, indicating that colonization of *E. coli* both in the SHIME gut and in mouse intestine gradually stabilized. However, a slower decline in *mCherry* gene abundance was found in the *E. coli* + ENR group, with the level being 1.12 times greater than that in the *E. coli* group of the SHIME system (Fig. 2A) and 1.53 times greater in the mice experiments (Fig. 2D), respectively. The same trend was observed for the *gfp* gene*.* The *gfp* abundance in the *E. coli* + ENR group was 1.24 times greater than that in the *E. coli* group of the SHIME system (Fig. 2B), and 2.07 times greater in the mice experiments (Fig. 2E). The same results were observed in the SHIME system of transverse colon and descending colon (Supplementary Fig. S1). These findings suggested that although *E. coli* colonization in both the SHIME and murine gut systems was generally inhibited, exposure to a low concentration of enrofloxacin promoted bacterial colonization in the gut.

An increased *gfp*/*mCherry* gene abundance ratio suggested a greater number of transconjugants, indicating horizontal gene transfer following the colonization of *E. coli* in the SHIME system and mouse gut. In the ascending colon samples of the SHIME system, the *gfp*/*mCherry* gene abundance ratio in the *E. coli* + ENR group was 1.10 times greater than that in the *E. coli* group (Fig. 2C). In the mouse intestine, the *gfp*/*mCherry* abundance ratio trend was consistent with that observed in the SHIME system, which in the *E. coli* + ENR group was 1.36 times greater than that in the *E. coli* group (Fig. 2F). Therefore, compared with *E. coli* exposure alone, low-concentration enrofloxacin exposure resulted in an increase in the *gfp*/*mCherry* abundance ratio.

**Figure 3:**
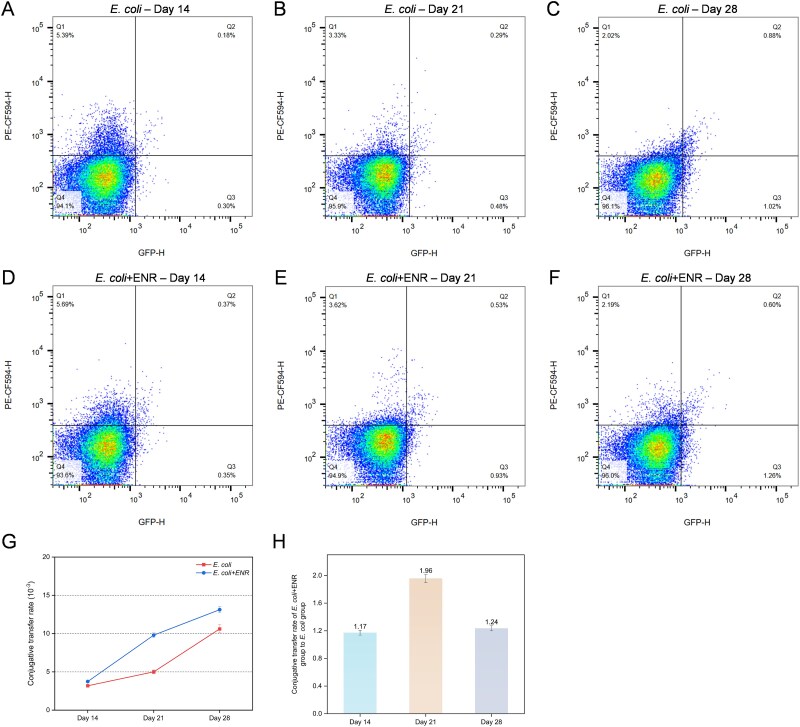
Quantification of conjugants (Q3) and potential recipient bacteria (nonfluorescent, Q4) in the mouse gut was performed using flow cytometry for the *E. coli* group (A–C) and the *E. coli* + ENR group (D–F). The conjugative transfer rate of the RP4 plasmid in the mouse gut was calculated as the ratio of conjugants (Q3) to the total population of potential recipient bacteria (nonfluorescent, Q4). Conjugative transfer rate of the RP4 plasmid in the mouse gut (G). The ratio of the conjugative transfer frequency in the *E. coli* + ENR group to the *E. coli* group (H).

Fluorescence microscopy and flow cytometry were used to further validate the conjugation transfer of plasmids *in vivo* mice experiments (Fig. 3A–F). The conjugation transfer rate was found to increase with the increasing exposure time, which ranged from 3.19 × 10^−3^ to 1.06 × 10^−2^ in the *E. coli* group and from 3.74 × 10^−3^ to 1.31 × 10^−2^ in the *E. coli + ENR* group (Fig. 3G)*.* The conjugation rate detected in this study was at the medium level of that found in previous studies [36, 37]. At Day 28, the conjugation transfer rate in the *E. coli + ENR* group was 1.24 times higher than that in the *E. coli* group (Fig. 3H). Consistent patterns were observed through fluorescence microscopy, where the number of fluorescent bacteria in the fecal microbiota of mice in the *E. coli* + ENR group was higher than that in the *E. coli* group on Day 28 (Supplementary Fig. S3). The presence of green fluorescent bacteria was dynamically tracked in the intestinal content samples of mice from the *E. coli* + ENR group at short gavage times (0, 2, 4, 6 h) (Supplementary Figs S4 and S5). Thus, the quantitative results of flow cytometry and the qualitative results of fluorescence microscopy demonstrated that a low dosage of enrofloxacin promoted the conjugation transfer of the resistance plasmids.

### Abundance and diversity of the simulated intestinal microbiota

**Figure 4:**
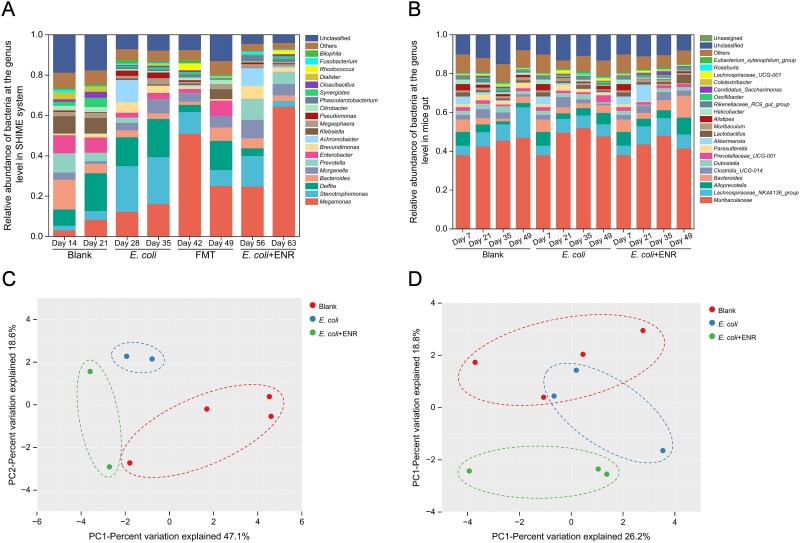
The bacteria composition detected in this study. Community composition of bacteria at the genus level in the ascending colon of the SHIME system (A) and mouse gut (B). Statistics were performed using one-way ANOVA with Benjamini–Hochberg correction for multiple comparisons. β-Diversity analysis using principal component analysis in the ascending colon of the SHIME system (C) and mouse gut (D).

The bacterial diversities and community compositions in mice (feces) and SHIME system (ascending colon) were investigated. At the genus level, the composition of gut microbiota in the *E. coli* + ENR group within the SHIME system differed from that in the Blank and *E. coli* groups, with a significant difference observed in the relative abundance of *Pseudomonas* (ANOVA: *P* = .015 < .05; Fig. 4A). *Delftia* was the dominant bacterial genus in the blank group (18.8%) and the *E. coli* group (18.9%), whereas *Megamonas* became the dominant bacterial genus in the *E. coli* + ENR group (Fig. 4A). At the phylum level, *Proteobacteria* was the dominant phylum in the Blank group (59.7%) and the *E. coli* group (73.4%), whereas *Firmicutes* became dominant in the *E. coli +* ENR group (69.2%) (Supplementary Fig. S6A). PCA revealed that the community structures of the Blank and *E. coli* groups presented high compositional similarity, whereas the *E. coli +* ENR group presented a distinct microbiome composition compared to the Blank and *E. coli* groups (Fig. 4C). The mouse gut community structure was more stable than that of the SHIME system, which was predominantly composed of *Bacteroidota* and *Firmicutes* at the phylum level (Supplementary Fig. S6B), and *Muribaculaceae* at the genus level with different proportions (Fig. 4B). PCA of the gut microbiota in mice revealed differences in microbial community composition between the *E. coli* + ENR group and the other two groups (*E. coli* group and Blank group) (Fig. 4D). In summary, exposure to *E. coli* alone did not significantly impact community structure, whereas additional enrofloxacin exposure led to significant changes. The same phenomenon is found in the intestinal microbiome of chickens using a prophylactic enrofloxacin dose of 75 mg/l through their drinking water [6].

**Figure 5:**
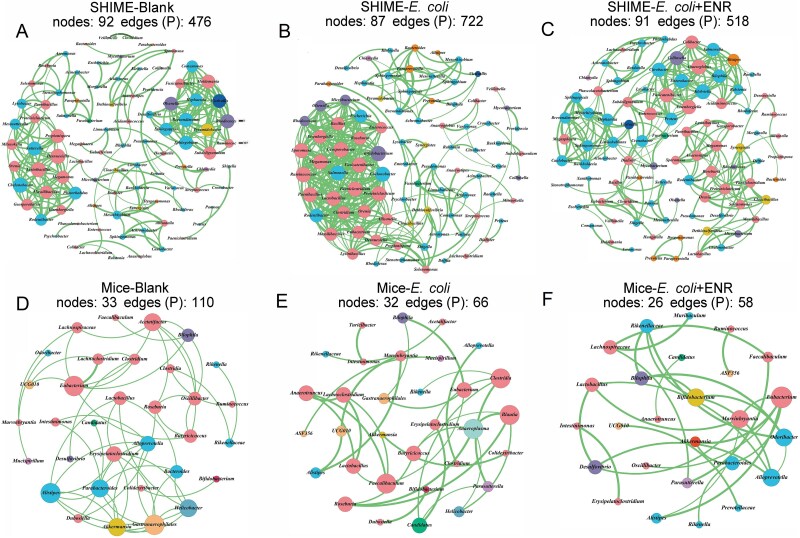
Correlation network analysis of the SHIME system and mouse gut microbiota. Each circle represents a bacterial species, with circle size indicating relative abundance and different colors representing different phyla. The significant positive correlations (*P* < 0.05) were shown in this figure. Thicker lines correspond to larger correlation coefficients.

Similarly, we defined Shannon’s diversity index as our measure of alpha diversity in this study because it accounted for both species’ richness and evenness among taxa. Compared with the Blank treatment, exposure to *E. coli* (ANOVA: *P* = .54) or enrofloxacin (ANOVA: *P* = .41) affected the diversity of gut microbiota in the SHIME system (Supplementary Fig. S7A). Enrofloxacin exposure resulted in the lowest Shannon diversity index at the genus level in the *E. coli* + ENR group (Blank group: 2.84; *E. coli* group: 2.42; *E. coli* + ENR group: 1.58). Exposure to *E. coli* led to a reduction in gut microbiota diversity, corresponding to the previously mentioned increase in the total relative abundance of nondominant phyla. Similarly, in the mouse intestine, the Shannon diversity index decreased from 2.58 to 2.27 in the *E. coli* + ENR group (Supplementary Fig. S7B). In total, lower species diversity was detected under combined exposure to enrofloxacin and *E. coli*. The correlation analysis among the simulated intestinal microbiota was further studied. In the mice experiment with enrofloxacin administration, correlation network analysis revealed that the number of edges was reduced by approximately 2-fold compared to the control group (Fig. 5). Especially, the number of edges was about two times lower in the mice experiments with the addition of enrofloxacin. In total, compared with the control group, the bacterial community exposed to enrofloxacin exhibited reduced network complexity, characterized by a decreased number of central nodes, fewer edges, and a lower average degree.

### Antibiotic resistance of the intestinal microbiota and the abundance of potential most likely bacterial hosts of antibiotic resistance genes

**Figure 6:**
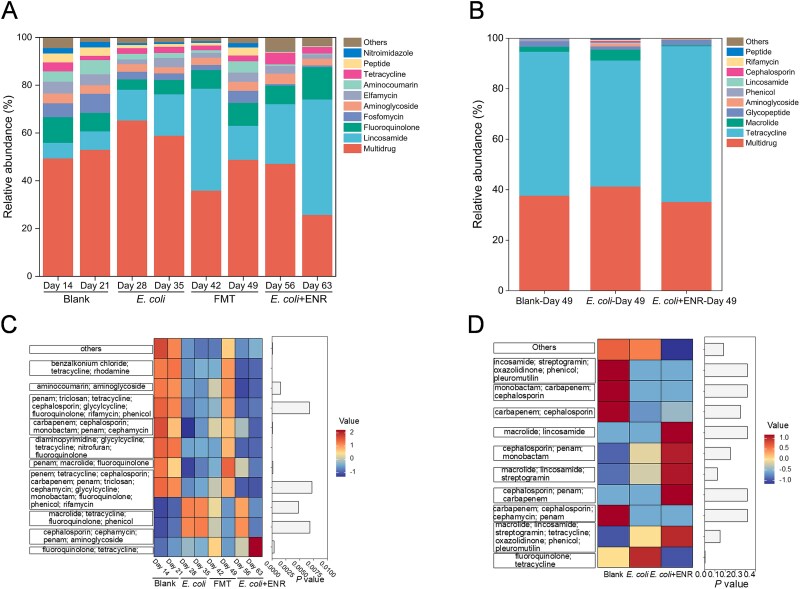
The ARGs detected in this study. The relative abundance of ARGs found in the ascending colon of the SHIME system (A) and mouse gut (B). Detailed classification of multidrug resistance within major categories of antibiotics in the ascending colon of the SHIME system (C) and mouse intestine (D).

The most significant change in antibiotic resistance within the SHIME system gut microbiota was observed for lincosamide antibiotics (Fig. 6A), which increased from 7.7% in the Blank group to 17.3% in the *E. coli* group and 48.3% in the *E. coli* + ENR group, followed by fluoroquinolone, which increased from 7.8% in the Blank group to 13.6% in the *E. coli* + ENR group. In contrast, multidrug resistance decreased from 53.1% in the Blank group to 25.8% in the *E. coli* + ENR group, with the most changes found for tetracycline–fluoroquinolone resistance (~3 times higher in the *E. coli* + ENR group than that in the Blank group) (Fig. 6C). Antibiotics are long considered to induce the propagation and spread of ARGs [38]. Enrofloxacin belongs to the fluoroquinolone antibiotic class. The increased proportion of genes conferring resistance to fluoroquinolone indicated that enrofloxacin exposure was the driving force behind the changes in mouse gut microbiota resistance. However, the complicated relationship between enrofloxacin exposure and various ARG production (e.g. lincosamide resistance) needed a more in-depth investigation. In the mouse gut microbiota, genes conferring resistance to tetracycline and multidrug antibiotics were predominant (Fig. 6B). The compositions of the ARGs in the different groups in the *in vivo* mice experiments were similar. Tetracycline–fluoroquinolone multidrug resistance peaked at 95.1% in the *E. coli* group, whereas carbapenem-cephalosporin-penam multidrug resistance was observed only in the *E. coli* + ENR group on Day 49 (Fig. 6D). Compared with those of the Blank and *E. coli* groups, distinct microbial resistance profiles were found in the *E. coli* + ENR group *in vitro* SHIME model experiments (Supplementary Fig. S8A) and *in vivo* mice experiments (Supplementary Fig. S8B).

**Figure 7:**
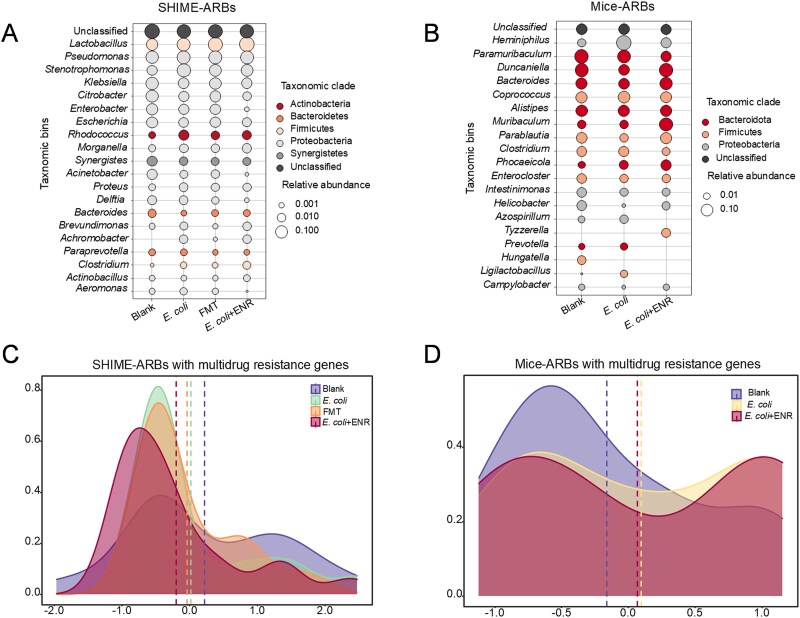
The ARG potential most likely bacterial hosts detected in this study. Community composition of ARG potential most likely bacterial hosts in the ascending colon in the SHIME system (A) and mouse gut (B). Density plot of the ARBs with multidrug resistance genes in the ascending colon of the SHIME system (C) and mouse gut (D). The *x*-axis represents the relative abundance changes of potential most likely bacterial hosts for multidrug resistance genes (ARGs) within the microbial community. The *y*-axis indicates data density, reflecting the probability density distribution of the corresponding *x*-axis values under different experimental conditions. The dashed lines in various colors represent the mean characteristic values of potential most likely bacterial hosts in each experimental group, illustrating the central tendency of the relative abundance distribution of potential most likely bacterial hosts for multidrug resistance genes.

The potential most likely bacterial hosts for ARGs in both *in vitro* SHIME model experiments and *in vivo* mice experiments were investigated in this study. The total absolute abundance of potential most likely bacterial hosts for ARGs in the *E. coli* + ENR group was 1.24 times greater than that detected in the *E. coli* group in the SHIME system and 4.65 times greater in the mice experiment, respectively (Supplementary Fig. S9). At the phylum level in the SHIME system, exposure to *E. coli* significantly increased the relative abundance of *Actinobacteria*, rising from 0.2% (*E. coli* group) to 3.0% (*E. coli* + ENR group) (independent-sample *t*-test: *P* = .026 < .05). At the genus level, coexposure to *E. coli* and enrofloxacin significantly decreased the relative abundance of *Enterobacter* compared to exposure to *E. coli* alone (independent-sample *t*-test: *P* = .020 < .05; Fig. 7A). The same was observed in the mice experiments (Fig. 7B). The density plot further confirmed the long distance between the group of Blank and *E. coli* + ENR both in the SHIME system (Fig. 7C) and mice experiments (Fig. 7D), indicating that exposure to enrofloxacin changed the composition of potential most likely bacterial hosts for ARGs.

### Concentrations of cytokines and enrofloxacin

The concentration of IL-8 ranged from 195 to 1456 pg/ml, followed by TNF-α (68 to 196 pg/ml), IL-1β (36 to 146 pg/ml), and IL-6 (14 to 54 pg/ml) (Supplementary Fig. S10). Among the different groups, the concentrations of the four pro-inflammatory factors in the *E. coli* group were 2.4–7 times higher than that detected in the Blank group and *E. coli* + ENR group.

Starting from Day 8 after the oral gavage of enrofloxacin, the concentration of enrofloxacin in mouse feces exhibited an increasing trend. Specifically, enrofloxacin concentrations in feces on Days 14, 21, and 28 were 1.62, 3.32, and 3.78 times higher than those on Day 8, respectively (Supplementary Fig. S11).

## Discussion

### Dietary exposure to enrofloxacin reduced the colonization resistance of the resident microbiota, thus promoting the colonization of *Escherichia coli* in the simulator of the human intestinal microbial ecosystem and mouse intestine

The colonization of exogenous bacteria on the human intestine is a prerequisite for their impact on human health. Enrofloxacin promoted the colonization of *E. coli* in the SHIME system and mouse intestine. The gut microbiota provides a barrier for the host to prevent the invasion and expansion of exogenous bacteria, a phenomenon known as colonization resistance. A wide variety of mechanisms are known to participate in colonization resistance [39]. Both the colonization resistance of the resident microbiota and the ability of exogenous bacteria to evade colonization resistance by virulence factors determine whether successful colonization can be achieved [40, 41]. In this study, only one exogenous bacterium, *E. coli*, was used to test colonization among the different groups. Thus, the ability to evade colonization resistance seemed to be the same across different groups. Therefore, the colonization resistance of the resident microbiota under *E. coli* exposure alone and under combined exposure to *E. coli* and enrofloxacin was assessed.

Microbial diversity has long been confirmed to affect host function [42, 43]. In this study, exposure to enrofloxacin reduced the species diversity of the gut microbiota. Communities with higher diversity are more resistant to potential invaders [44]. In addition, antibiotic treatment often leads to the loss of diversity and promotes the invasion of alien species [45, 46]. The decrease in microbial diversity in the small and large intestines caused by antibiotics promotes chronic infection of *Clostridium difficile* [47], indicating that a decrease in microbial diversity is beneficial for the colonization of exogenous bacteria. The successful invasion of exogenous bacteria reduces the richness of the resident microbiota, which is detrimental to the resident microbiota. Therefore, dietary exposure to enrofloxacin reduces the species diversity of the gut microbiota and promotes the colonization of *E. coli*.

In addition to microbial diversity, the interaction strengths among species influence the colonization of exogenous bacteria. On the one hand, the colonization resistance increases when resident microorganisms interact strongly with each other [48]. Under external enrofloxacin exposure, the resident microbiota established incompact interrelations, resulting in decreased colonization resistance. In contrast, the interaction strengths between the resident microbiota and exogenous bacteria also determine whether exogenous bacteria can successfully colonize. Most symbiotic bacteria have weak antagonistic effects on exogenous species, and the presence of certain key species can significantly increase colonization resistance. For example, *Enterococcus faecalis* has a very significant inhibitory effect on *Enterococcus faecium* [49]. Bacteria at the same phylum level tend to occupy similar ecological niches, leading to intense competition and increased colonization resistance [50]. The *E. coli* species selected in this study belongs to *Proteobacteria*, and the abundance of *Proteobacteria* decreased after enrofloxacin exposure (Supplementary Fig. S6), thereby reducing colonization resistance to *E. coli*.

Cytokines play crucial roles in the immune system and can respond to the body’s inflammatory response. The body not only repairs oxidative damage through the inflammatory response but also releases large amounts of immune cytokines to clear infection when resisting foreign invasion or clearing abnormalities [51]. The concentrations of four pro-inflammatory factors, IL-6, IL-8, IL-1β, and TNF-α, under combined exposure to *E. coli* and enrofloxacin, were 0.49, 0.64, 0.74, and 0.80 times lower than those detected under exposure to *E. coli* alone. There is no consensus on the inflammatory response caused by antibiotic exposure. Oral antibiotics induce the translocation of live indigenous commensal bacteria in the colonic epithelium and promote the inflammatory response [52]. In another study, the antibacterial effect of ciprofloxacin reduces the intestinal toxicity caused by *Shigella flexneri*, and lower concentrations of IL-1β and IFN-γ are detected in the treatment groups than in the controls [53]. These findings are consistent with other evidence that treatment of mice with broad-spectrum antibiotics prevents increases in stressor-induced cytokines [54–56]. The intestinal microbiota induces resident macrophages to convert pro-IL-1β into IL-1β, increasing its concentration and making the cell’s response to bacteria more rapid, thus clarifying the pathogenesis of neutrophils [57]. The lower concentration detected in this study indicated that dietary exposure weakened the immune response of the body when exposed to external bacterial invasion and thus reduced colonization resistance to *E. coli*.

Simulation of the SHIME model is considered a valuable *in vitro* research tool for elucidating the direct effects of antibiotics on the human gut microbiota, free from neurohumoral regulation, individual differences, dietary habits, and physiological states [23]. The mice experiments make up for the lack of immune regulation *in vitro* experiments [58, 59]. In our study, mice and SHIME reached the same conclusion, indicating that both SHIME and mice experiments could simulate the effects of long-term dietary intake of antibiotics on the resistance of human gut microbiota. In total, dietary exposure to enrofloxacin antibiotics weakened the colonization resistance by reducing microbial diversity, strengthening interactions, and weakening immune response of resident microbiota, and promoted the colonization of exogenous *E. coli* in the simulated human gut (Fig. 5).

### Dietary exposure to enrofloxacin promoted the horizontal transfer of resistance plasmids and changed the simulated human gut antibiotic resistome

The spread of plasmids among different bacteria plays a significant role in the evolution of antibiotic resistance in bacteria, as most known resistance genes have been detected on plasmids [60]. The *gfp*/*mCherry* ratio in the mouse gut under combined exposure to *E. coli* and enrofloxacin was approximately 1.24 times higher than under *E. coli* exposure alone, suggesting that enrofloxacin facilitates horizontal gene transfer. Metagenomic sequencing was used to further investigate the effects of dietary exposure to enrofloxacin on antimicrobial resistance in simulated human gut bacteria. The results revealed that the abundance of the potential most likely bacterial hosts of ARGs in the *E. coli* + ENR group was ~4.65 times greater than that in the *E. coli* group, indicating that enrofloxacin exposure promoted the growth of potential ARBs, which confirmed the theory of fitness cost. Fitness cost is defined as ARGs imposing a general burden to the resistant organism in an antibiotic-free environment, and thus susceptible strains have a growth advantage [61, 62]. In an environment with antibiotics, the growth rates of ARBs exceed those of antibiotic-sensitive strains, thus exhibiting growth advantages [63]. The absolute abundance of potential most likely bacterial hosts of ARGs subsequently increased over time.

In addition to the vertical transmission of ARGs caused by the growth advantages of ARBs, chromosomal mutations and horizontal gene transfer are important factors leading to the proliferation of ARGs [64, 65]. Compared with chromosomal mutations, lower costs of obtaining ARGs mediated by horizontal gene transfer are needed [66, 67]. Therefore, horizontal transfer has become the main reason for the current acquisition of multidrug resistance in bacteria. The RP4 conjugation transfer undergoes a continuous process of replication, allocation, and binding, which is controlled by the mating pair formation (Mpf) system [68]. The abundance of the Mpf system key gene *traF* in the *E. coli* + ENR group was 1.42 times higher than that in the *E. coli* group of *in vitro* SHIME experiments and 2.19 times that of the *in vivo* mice experiments (Supplementary Fig. S12A and B), which further indicated that enrofloxacin could regulate Mpf systems to promote the conjugation transfer of plasmid. This was also suggested by the expression of genes related to the type IV secretion system (Supplementary Fig. S12C and D). The T4SS is a multifunctional, multicomponent transmembrane channel structure that can pass both proteins and DNA [69]; thus, joint transfer mediated by the T4SS is one of the important mechanisms of horizontal gene transfer. Most conjugative plasmids and integrated conjugative elements rely on their own encoded T4SS for conjugation and transfer [70]. Therefore, these changes under enrofloxacin exposure induced the conjugation transfer of the RP4 plasmid into the gut microbiota*.*

**Figure 8:**
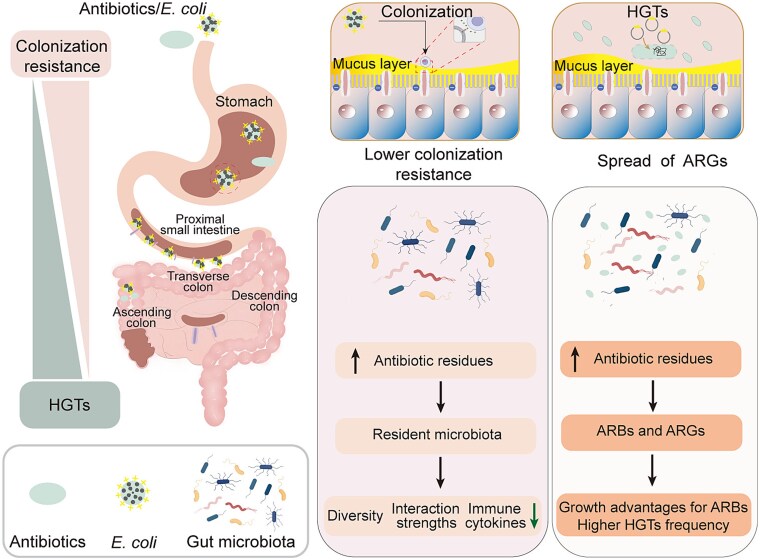
Possible mechanisms of dietary exposure to veterinary antibiotics affect the colonization of exogenous bacteria in the human intestine and spread of ARGs.

Although the horizontal gene transfer of ARGs was promoted under both the *E. coli* exposure alone and combined exposure to *E. coli* and enrofloxacin, it is currently unknown whether the horizontal transfer of exogenous ARGs leads to changes in the antibiotic resistance of the human gut microbiota. Our results showed that the bacterial community and ARGs exhibited the farthest distance between the group under combined exposure to *E. coli* and enrofloxacin and the other experimental groups. However, the groups exposed to *E. coli* alone and the controls were clustered, indicating that their bacterial communities and ARG compositions were similar. Both *in vitro* SHIME model experiments and *in vivo* mice experiments confirmed that *E. coli* exposure alone had little effect on the simulated human gut microbiota and antimicrobial resistance. Based on the results mentioned above, the antibiotic resistance of the gut microbiota changed significantly with the increasing growth advantages of potential ARBs and the frequency of horizontal gene transfer under dietary exposure to enrofloxacin (Fig. 8). Antibiotic treatment (ciprofloxacin, cefuroxime, doxycycline, and azithromycin) in 10 healthy adult volunteers aged 18–65 years lead to persistent expansion of ARGs in the human gut microbiota [71]. The same results have also been reported in other studies, which confirm the accumulation of resistance in the human gut microbiota by following exposure to antibiotics [72–74]. In this study, residues of enrofloxacin in food were found to induce the proliferation of ARGs in the simulated human gut microbiota. Although maximum residue limits are considered to pose no health risk to the consumer and have no effect on the production process, they do not consider whether the permissible residues cause antimicrobial resistance [75]. Therefore, more attention should be paid to the enrofloxacin residues in food.

### Intestinal microbiota and antibiotic resistome were significantly time dependent upon enrofloxacin exposure

Conventional antibiotic treatment for humans has generally been limited to short periods of time, such as <7 days [76, 77]. However, dietary exposure to antibiotics generally lasts longer because changes do not often occur once a person’s purchasing and dietary habits are formed. In this study, enrofloxacin exposure lasted for 2 weeks in the SHIME system and 6 weeks in the mice experiments. In the absence of exposure to *E. coli* and enrofloxacin, the diversity and composition of the microbiome and the antibiotic-resistant genome did not change significantly over time, confirming the stability, resilience, and robustness of the gut microbiome in healthy adults [78, 79]. As the duration of enrofloxacin exposure increased, the relative abundance of *Proteobacteria* decreased from 49.4% on Day 56 to 14.3% on Day 63 in the *E. coli* + ENR group. The relative abundances of *Firmicutes* in the *E. coli* + ENR group on Day 56 and Day 63 were 2.87- and 4.16-fold higher, respectively, than that in the Blank group on Day 21. Additionally, the Shannon index of the SHIME system tended to decrease with prolonged enrofloxacin exposure, decreasing from 1.16 on Day 56 to 1.01 on Day 63 at low enrofloxacin concentrations. The results of the 4-week enrofloxacin exposure *in vivo* mice experiment also revealed the same trends in terms of bacterial community and Shannon index, indicating that the gut microbiota structure in the simulated human gut significantly changed in a time-dependent manner under enrofloxacin exposure. Enrofloxacin exposure resulted in a time-dependent difference in gut microbiota β-diversity, with the greatest dissimilarity observed after prolonged exposure (Supplementary Fig. S13).

The antibiotic resistomes were compared under different exposure times. The absolute abundance of potential most likely bacterial hosts of ARGs on Day 63 (exposure time of 14 days) was 1.45 times higher than that detected on Day 56 (exposure time of 7 days) in the *in vitro* SHIME model experiments. The ARGs also changed with prolonged enrofloxacin exposure. The relative abundance of genes conferring resistance to fluoroquinolone on Day 63 (exposure time of 14 days) was 2.53 times higher than that detected on Day 56 (exposure time of 7 days). Moreover, tetracycline and fluoroquinolone resistance showed drastic changes in the *E. coli* + ENR group, rising from 23.0% (Day 21, control group) to 62.4% on Day 63 of enrofloxacin exposure. Although changes at the genetic level varied across individuals, a targeted response to enrofloxacin was found in this study. The same results were also confirmed after prophylactic antibiotic treatment in chickens, which concluded that prolonged exposure to antibiotics increases the abundance of ARGs in the chicken intestine [6]. The resistance to beta-lactam antibiotics is the most affected, suggesting that there is a targeted response to amoxicillin [80].

In conclusion, the effects of dietary exposure to enrofloxacin on both the intestinal microbiota and the antibiotic resistome were obviously time-dependent. Our results provided strong evidence that intestinal microbiota significantly changed and that antibiotic resistance increased with increasing exposure time. Antibiotic residues in food have been confirmed to be harmful to humans [75, 81]. Meanwhile, the concentration, detection, and removal of antibiotics in food have become a hot topic of research [3, 82]. The impact of antibiotics is cumulative in the human body [83]. Several studies also indicated the persistent long-term effects of antibiotic exposure on the human gut microbiota and even posttreatment [80, 84, 85]. However, these previous studies did not explain what happens in the human intestine through dietary veterinary antibiotic exposure. The SHIME system can simulate the human gastrointestinal system. In our SHIME system experiment, we added enrofloxacin to the anterior end of the stomach. In mouse experiments, the enrofloxacin was administered orally. In addition, the concentration of enrofloxacin we selected is based on the residual concentration in food as exhibited in Section “Antibiotic, strains, and culture conditions.” Therefore, our results might provide relevant evidence that dietary intake of veterinary antibiotics may induce the ARG transmission from food to the human gut by promoting the colonization of ARBs and horizontal transfer of resistance plasmid. Alarmingly, the duration of exposure to foodborne antibiotic residues was pivotal among these processes. The results of this study are of great significance for establishing a more accurate and efficient food safety and risk assessment system and maintaining public health and well-being.

A major limitation of our study was only one veterinary antibiotic, enrofloxacin, was used in this study to investigate the horizontal transfer of resistance plasmids, which limited the generalizability of our findings to other veterinary antibiotics commonly used in livestock. Different antibiotics may confer varying effects on gut microbiota and ARG dynamics. In addition, there are still limitations to revealing human biology in mouse models. In the mouse biological system, especially the immune system, is not completely consistent with humans. Therefore, further studies will be needed to investigate the impact of more veterinary antibiotics on human gut microbiota resistance combined with molecular simulation methods.

## Supplementary Material

ISMEJ-D-24-01301R5-Supplementary_Materials_wraf045

## Data Availability

Authors can confirm that all relevant data are included in the paper and/or its supplementary materials file. The online version contains supplementary information available at http://doi.org/10.1093/ismejo/wraf045. 16S rRNA gene amplicon and metagenome sequence data are available at the US National Center for Biotechnology Information (NCBI; Bethesda, MD, USA; Accession No. PRJNA1197106).
