# Supplementary material for: Dietary intake of enrofloxacin promotes the spread of antibiotic resistance from food to simulated human gut
Source: ISME J. 2025 Mar 23;20(1):wraf045. doi: 10.1093/ismejo/wraf045 (PMC12825312; doi:10.1093/ismejo/wraf045)
Supplement: ISMEJ-D-24-01301R5-Supplementary_Materials_wraf045 [file ismej-d-24-01301r5-supplementary_materials_wraf045.pdf]

Supplementary Material

Dietary intake of enrofloxacin promotes the spread of antibiotic resistance from food to simulated human gut

Contents

Supplementary Texts

Text S1 The construction of the SHIME system

Text S2 *In vivo* mice experiments

Text S3 High-throughput sequencing of 16S rRNA genes

Text S4 Metagenomic sequencing analysis

Text S5 Identification of ARG potential hosts

Text S6 Detailed method for extracting bacteria from mouse feces

Text S7 Upright fluorescence microscope and flow cytometry

Text S8 Detection of cytokines

Text S9 Detection of enrofloxacin

Text S10 Code for correlation network analysis

Supplementary Tables

Table S1 The composition of the SHIME culture

Table S2 The composition of the pancreatic juice

Table S3 Grouping and feeding of mice

Table S4 Primers used for fluorescence quantitative PCR

Table S5 Fluorescence quantitative PCR reaction system

Table S6 Statistics of the results of non-redundant gene sets *in vitro* SHIME experiments

Table S7 Statistics of the results of non-redundant gene sets *in vivo* mice experiments

Table S8 The standard curve equations established in this study for the quantification of cytokines

Table S9 Mass spectrometry parameter information

Supplementary Figures

Figure S1 The abundance of *gfp* and *mCherry* (log10 transfer) in the SHIME system of transverse colon and descending colon. Logarithmic abundances of *mCherry* (**A**) and *gfp* (**B**), and the logarithmic abundance ratio of *gfp* gene to *mCherry* gene (**C**) in the transverse colon of the SHIME system. Logarithmic abundances of *mCherry* (**D**) and *gfp* (**E**), and the logarithmic abundance ratio of *gfp* gene to *mCherry* gene (**F**) in the descending colon of the SHIME system.

Figure S2 The gating strategy of sorting the *gfp*-expressing cells from mating mixtures by a cell sorter (FACSAria™ Fusion, BD, USA). UsingFlowJo_10.8.1 software for data analysis. The procedure consists of four consecutive gates with bivariate plots: Gate I was drawn to select only particles of bacterial sizes based on forward and side scatter (SSC-A vs FSC-A) (A); Gates II and III were drawn to remove any doublets and select singular bacterial cells using (SSC-W vs SSC-A) and (FSC-H vs FSC-A), respectively (B-C); Gate IV was analyzed using GFP-H vsPE-CF594-H (*mCherry*) fluorescence intensity, to distinguish between the donor (*mCherry*, Q1), recipient (no fluorescence, Q4), and transconjugant cells (*gfp*, Q3) (D).

Figure S3 Fluorescence microscopy images of fecal bacterial *in vivo* mice experiments. The red-fluorescent bacteria were the donor *E. coli* labelled with a *mCherry* gene and the green fluorescent bacteria were transconjugants with newly acquired *gfp*-tagged RP4 plasmid. *E. coli* group (**A**) and *E. coli*+ENR group (**B**).

Figure S4 Fluorescence microscopy presents the dynamic process of red-fluorescent bacteria (the donor *E. coli* labelled with a *mCherry* gene) and green-fluorescent bacteria (transconjugants with newly acquired *gfp*-tagged RP4 plasmid) in the intestinal contents of mice at different time points of the first gavage in *E. coli* group. 0 h (**A**), 2 h (**B**), 4 h (**C**) and 6 h (**D**).

Figure S5 Fluorescence microscopy presents the dynamic process of red-fluorescent bacteria (the donor *E. coli* labelled with a *mCherry* gene) and green-fluorescent bacteria (transconjugants with newly acquired *gfp*-tagged RP4 plasmid) in the intestinal contents of mice at different time points of the first gavage in *E. coli*+ENR group mice. 0 h (**A**), 2 h (**B**), 4 h (**C**) and 6 h (**D**).

Figure S6 The bacteria composition detected in this study. A-B Community composition of gut bacteria at the phylum level. Ascending colon of the SHIME system (**A**) and mouse gut (**B**).

Figure S7 A-B Alpha diversity analysis of gut microbiota was conducted using the Shannon index to measure species richness and diversity. Ascending colon of the SHIME system (**A**) and mouse gut (**B**).

Figure S8 Hierarchical clustering analysis of ARGs detected in the SHIME system (**A**) and mouse gut (**B**).

Figure S9 The absolute abundance of potential hosts of ARGs detected in the SHIME system (**A**) and mice experiments (**B**).

Figure S10 The concentration of four proinflammatory factors in the gut contents of mice. Chronic low-dose enrofloxacin exposure reduces the expression of pro-inflammatory cytokines IL-6, IL-8, IL-1β, and TNF-α in the gut microbiota. IL-6 (**A**), IL-8 (**B**), IL-1β (**C**), and TNF-α (**D**).

Figure S11 The concentration of enrofloxacin detected in the mice feces.

Figure S12 The abundance of *traF* detected *in vitro* SHIME experiments (**A**) and *in vivo* mice experiments (**B**); Heatmap analysis of the abundance of type IV secretion system-related genes *in vitro* SHIME experiments (**C**) and *in vivo* mice experiments (**D**).

Figure S13 Beta diversity of gut microbiota at the genus level for all samples was assessed based on Bray-Curtis dissimilarity. In the bubble plot, the size of the circles represents the magnitude of the Bray-Curtis dissimilarity value; larger circles indicate greater beta diversity differences between samples. Ascending colon of the SHIME system (**A**) and mouse gut (**B**).

Text S1 The construction of the SHIME system

As shown in Figure 1A, five glass reactors, which simulate the stomach, small intestine, ascending colon, transverse colon and descending colon, were included in the SHIME simulator. A constant-temperature circulator was used to maintain the temperature at 37 °C. To simulate the peristalsis of the gastrointestinal tract, a magnetic stirrer was applied. The compositions of the SHIME culture and pancreatic juice are listed in Table S1 and Table S2, respectively. Both the SHIME culture and the pancreatic juice were sterilized at 121 °C for 30 minutes before use. The pH values of the ascending colon, transverse colon, and descending colon were maintained between 5.5-5.9, 6.0-6.4, and 6.5-6.9, respectively, by a pH controller. The three sections of the colon were placed in a light-proof fume hood, and nitrogen gas was injected for 15 minutes every 8 hours during the culture period to maintain an anaerobic environment. Fifty milliliters of feces diluted from healthy volunteers (healthy hosts who had not used antibiotic drugs in the past six months) were inoculated into the ascending colon, transverse colon and descending colon.

Table S1 The composition of the SHIME culture

Name	Content (g)	Name	Content (g)
D-Glucose anhydrous	0.4	yeast extract	3.0
arabinogalactan	1.0	peptone	1.0
L-Cysteine hydrochloride	0.5	mucin	1.0
soluble starch	4.0	pectin	2.0
Xylan	1.0		

Table S2 The composition of the pancreatic juice

Name	Content (g)
Pig bile salt	6.0
pancreatin	0.9
sodium bicarbonate	12.5

To stimulate human eating habits, 200 mL of SHIME culture is added to the simulated stomach every 8 hours (7:00, 15:00, 23:00 every day) and fermented for 2 hours in the gastric simulation tank. Then, all the food is pumped into the small intestine simulation tank. At the same time, 100 mL of pancreatic juice is added to the simulated small intestine, and all the feed liquid is transferred to the ascending colon reactor after 4 hours. Afterwards, the feed liquid is continuously and uniformly pumped from the ascending colon to the transverse colon through the peristaltic pump and then pumped from the transverse colon to the descending colon. The feed liquid in the descending colon is finally pumped into the waste liquid tank. The whole process simulates the daily eating and excretion of human beings.

In this study, the complete experimental cycle included 9 weeks. A total of 4 periods, namely, the Blank, *E. coli*, FMT and *E. coli*+ENR periods, were conducted. The specific experimental process is shown in Figure 1A. First, only the SHIME special medium was added to the SHIME system for 3 weeks to stabilize the intestinal microbial community (Blank group). Then, approximately 10^{10} CFU of *Escherichia coli* K-12 MG1655 (*E. coli*) were added into the system on the 22nd day of the experiment and maintained 2 weeks of cultivation (*E. coli* group). Fifty milliliters of the same healthy host’s feces mixture was inoculated into the colon to perform faecal bacteria transplantation, and the samples were allowed to recover for 2 weeks (FMT group). Then, 10^{10} CFU *E. coli* were added to the SHIME medium for cultivation for another 2 weeks, with the addition of 70 μg of enrofloxacin per day (*E. coli*+ENR group).

On each sampling day, 15 mL samples were collected from the ascending, transverse, and descending colon in sterile centrifuge tubes at three time points prior to each meal, and finally the three samples collected from the same colon reactor were mixed into a single sample and placed into sterile centrifuge tubes on an ultra-clean bench. Afterwards, the samples were preserved with 30 % glycerol and then placed in a -20 °C freezer for use in subsequent experiments.

Table S3 Grouping and feeding of mice

Group	*Escherichia coli* K-12 MG1655	Enrofloxacin
Blank	/	/
E. coli	10^9 CFU/mL	/
E. coli+ENR	10^9 CFU/mL	0.8 mg/day

Text S2 *In vivo* mice experiments

After one week of adaptive feeding, 36 mice were numbered and randomly divided into 3 treatment groups. Each group had 2 replicates, with 6 mice in each replicate. Conventional feeding conditions were employed, which included a temperature of 25±1 °C and a relative humidity of 55±5%. The gavage method was used for the infusion of *E. coli* and enrofloxacin. Each group of mice was orally administered at 8:00 am every day.

During the experiment, the faeces of the mice were collected from 9:00 am to 10:00 am on Days 7, 14, 21, 28, 35, 42 and 49. The faeces collected from the same group were mixed into one sample and stored at -80 °C for further analysis. On Days 7, 21, 35, and 49, the faeces were collected for high-throughput 16S rRNA gene sequencing. Metagenomic sequencing was performed on faecal samples collected on Day 49. To study the immune regulation of mice, the intestinal jejunum to ileum segments were collected after the mice were sacrificed by cervical dislocation.

Text S3 High-throughput sequencing of 16S rRNA genes

(1) Experimental procedure

1) DNA extraction, followed by Nanodrop quantification.

2) Design primers targeting conserved regions within the sequences, incorporating sample-specific Barcode sequences. PCR amplification is then performed on the variable regions of the rRNA gene (either individual or consecutive multiple regions) or specific gene fragments.

3) PCR products are purified and recovered using magnetic beads.

4) The recovered PCR amplification products are quantified fluorescence. Based on the quantification results, samples are pooled in the required proportion according to the sequencing depth for each sample.

5) Library preparation is conducted using the VAHTS Universal DNA Library Prep Kit for Illumina V3 and the VAHTS DNA Adapters set 3-set 6 for Illumina, provided by Vazyme Biotech Co.,Ltd.

6) The libraries undergo quality control, and only those passing QC are subjected to high-throughput sequencing on the NovaSeq 6000 sequencing platform.

(2) Analysis procedure

1) The raw sequences that pass quality filtering are divided into libraries and samples based on index and Barcode information. Barcode sequences and primers are removed.

2) Sequence denoising is performed according to the QIIME2 dada2 pipeline.

3) The taxonomic composition of each sample (or group) at different taxonomic levels is visualized to gain an overview of the overall structure.

4) Alpha diversity levels for each sample are assessed based on the distribution of ASVs across different samples. Sequencing depth adequacy is evaluated using rarefaction curves.

5) At the ASV level, distance matrices are calculated for each sample, and beta diversity differences between samples (or groups) are assessed through various unsupervised ordination and clustering methods.

6) Biomarker with statistically significant differences between groups are identified through inter-group differential analysis.

7) Correlation and association analyses are conducted to explore the relationship between microbial communities and environmental factors.

8) Functional prediction analysis is performed to predict gene functions or phenotypes.

Table S4 Primers used for fluorescence quantitative PCR

Gene	Primers	Sequence (5’-3’)	Annealing temperature
gfp	F	TCCGTTCAACTAGCAGACCAT	60 ℃
	R	TCATCCATGCCATGTGTA ATCC	
mCherry	F	ACGGCGAGTTCATCTACA	60 ℃

	R	GAGGTGATGTCCAACTTGAT	
16S rRNA	F	CGGTGAATACGTTCYCGG	55 ℃
	R	GGWTACCTTGTTACGACTT	

Table S5 Fluorescence quantitative PCR reaction system

Process	Temperature	Time	
Denaturation	94 ℃	30 s	
	94 ℃	5 s	40 cycles
Annealing	The annealing temperature for specific genes (Table S4)	15 s	
Extension	72 ℃	10 s	

Text S4 Metagenomic sequencing analysis

(1) Processing of original sequence

The raw sequences obtained from sequencing typically contain low-quality reads. To ensure subsequent analyses' accuracy, raw reads must undergo quality filtering to generate clean reads. The filtering process includes the following steps:

1) Raw tags were processed using the fastp software (version v0.23.1.1), with the parameters -5 -W 50 -M 20 -1 60 -n 0 -g -A applied to retain high-quality sequencing data (clean tags). Specifically, "-5" indicates quality control from the 5' end using a sliding window, with sequences below the threshold being discarded; "-W" specifies the window size; "-M" sets the average quality score within the window; "-1" defines the minimum length threshold for sequence retention; "-n" sets the maximum allowable number of N bases in a sequence, with sequences exceeding this value being discarded; "-g" trims polyG tails; and "-A" ensures that adapters are not removed.

2) The host genome sequence was used as a reference for alignment with the sample reads using Bowtie2 (version v2.2). The alignment was performed with the following parameters: --seed 123456, -I 200, -X 10000, and --un-conc-gz, to remove host-derived contamination. Cleaned read data were obtained after quality control of sequencing results. Specifically, the "--seed" parameter was set to a fixed random seed to ensure consistency across analyses; the "-I" and "-X" parameters were used to specify the minimum and maximum fragment lengths, respectively; and the "--un-conc-gz" parameter ensured that sequences not aligning with the reference genome were outputted.

(2) Metagenomic Assembly

Metagenomic assembly was performed using the software MEGAHIT (version v1.1.2 with default parameters), with contigs shorter than 300 bp filtered out. The assembly results were evaluated using QUAST (version v2.3 with default parameters).

(3) Metagenomic Component Analysis

Gene identification in the metagenomes was carried out using MetaGeneMark (version v3.26 with default parameters) (http://exon.gatech.edu/meta_gmhmmp.cgi, Version 3.26), with default settings (parameters -A -D -fG) to identify coding regions in the genomes.

(4) Construction of a non-redundant gene set

Redundancy was removed using MMseqs2 software (https://github.com/soedinglab/mmseqs2, Version 11-e1a1c), with a similarity threshold of 95% and a coverage threshold of 90%.

(5) Gene annotation

Metagenomic function annotation included Non-Redundant Protein Database (Nr) annotation, Kyoto Encyclopedia of Genes and Genomes (KEGG) database annotation, Comprehensive antibiotic research database (CARD) annotation, Virulence Factor database (VFDB) annotation. Annotation details are shown in the following database modules:

Non-redundant protein database (Nr) annotation: In the specific annotation, the protein sequence of the non-redundant genes was compared with the Nr database by Blast (diamond v0.9.29.130 comparison screening threshold E-value 1e-5) to find the most similar sequence in the Nr database. The corresponding annotation information of the sequence was the annotation information of the corresponding sequenced genome gene.

KEGG is a comprehensive database that integrates genomic, pathway, and compound information for biological organisms. The database performs sequence clustering analysis, forms orthologous protein groups, and assigns distinct KO (KEGG Orthology) sequences to different protein groups. In the annotation process, the protein sequences of non-redundant genes are subjected to BLAST comparison with the sequences recorded in the KEGG database (BLAST is performed using DIAMOND v0.9.29, with a threshold E-value of 1e-5). The most similar sequence found in the KEGG database is identified, and its annotation information, corresponding KO number, and the position within the relevant pathway of the KO are assigned as annotation information, KO number, and position within the biological process pathway for the corresponding gene in the sequenced genome.

Comprehensive antibiotic research database (CARD) annotation: In the specific annotation, the software rgi (version 4.2.2 default Perfect, Stric algorithm) in CARD database was used to compare the protein sequences of non-redundant genes in the database, find out the corresponding sequences in the database on the comparison, and obtain the corresponding resistance genes and resistance-related information.

VFDB (Virulence Factor Database) is a comprehensive collection of well-characterized virulence factors from bacterial pathogens. These virulence factors help pathogens establish infections in hosts, evading the host's immune system, surviving within the host, and causing diseases. The database contains two datasets of virulence factor sequences: one is the experimentally validated core dataset, set A, and the other is the full dataset, set B, which includes both the experimentally confirmed virulence factors from set A and predicted virulence factors. This database can help predict potential virulence factors in bacterial genomes that have not yet been extensively studied. For specific annotation, the protein sequences of non-redundant genes are aligned against the core dataset set A using BLAST (BLAST 2.2.31+, default parameters, E-value threshold of 1e-5). The most similar sequence found in the database provides the relevant annotation information for the corresponding gene in the sequenced genome.

(6) Relative abundance of microbial genomes among individuals

The relative abundance of genes was determined by constructing non-redundant gene sets. Redundant genes were removed using the MMseqs2 software (https://github.com/soedinglab/mmseqs2, Version 11-e1a1c), with a protein sequence similarity threshold set at 95% and a coverage threshold at 90%. The abundance of functional genes was then calculated based on gene counts obtained from non-redundant gene sets. Genome coverage was assessed through a unique comparison of Illumina reads, with normalization to 1 Gb of sequence data to account for differences in sequencing depth across individuals. The total coverage of all species in the non-redundant bacterial genome set for each individual was summed, and the relative abundance of each species was calculated as a proportion of this total.

Table S6 Statistics of the results of non-redundant gene sets *in vitro* SHIME experiments

Type	Gene set number	Total length(bp)	Average(bp)	Max length(bp)	Min length(bp)
Gene set	515,182	361,305,483	701	42,918	102

Table S7 Statistics of the results of non-redundant gene sets *in vivo* mice experiments

Type	Gene set number	Total length(bp)	Average(bp)	Max length(bp)	Min length(bp)
Gene set	734,650	485,910,496	661	47,253	32

Text S5 Identification of ARG potential hosts

MEGAN was used to annotate all open reading frames (ORFs) on contigs carrying ARGs, and then the R Studio was used to vote on the annotation results. If more than 50% of ORFs on a contig were assigned to the same taxon, the annotation results at this classification level were determined to be the host of the contig. The sequences extracted from contigs carrying multiple sulfonamide resistance genes were aligned with NCBI by BLASTN, and species annotation was performed at the genus level.

Text S6 Detailed method for extracting bacteria from mouse feces

(1) Sample collection: Fecal samples from mice were collected and immediately placed in sterile containers. All procedures were conducted under sterile conditions, and samples were promptly transferred to 4°C refrigerators.

(2) Sample preprocessing: Fecal samples were physically homogenized using a homogenizer or grinder to ensure thorough disruption. During grinding, cold PBS or liquid nitrogen was added as needed to prevent bacterial inactivation due to heat exposure.

(3) Sample dilution: The homogenized fecal samples were dissolved in sterile phosphate-buffered saline (PBS) at a ratio of 5 mL PBS per gram of sample. The mixture was thoroughly homogenized using a homogenizer to ensure even distribution, facilitating subsequent bacterial extraction.

(4) Sample clarification: Large particle impurities and non-bacterial components were removed by low-speed centrifugation (1000 rpm for 5 minutes). The supernatant was collected for further processing. The centrifugation process was performed at 4°C to prevent bacterial heat-induced damage.

(5) Bacterial isolation: Appropriate nylon filter membranes with a defined pore size were cut, and filtration was performed using a purification filter. The first filtration step employed a large-pore (1 μm) filter to remove larger particles, while the second step used a 0.22 μm filter for bacterial isolation. The filtrate was collected for the next step.

(6) Centrifugal collection: Medium-speed centrifugation (3000 rpm for 10 minutes) was used to further separate bacteria. The bacterial pellets were collected following centrifugation. Temperature during centrifugation was maintained at 4°C to avoid bacterial damage.

(7) Bacterial collection: The bacterial pellet was resuspended in PBS, and a double-layer filter with a 0.22 μm pore size was used for further purification. The filtered bacterial solution was collected and stored under sterile conditions for subsequent analysis.

Text S7 Upright fluorescence microscope and flow cytometry

A fluorescence microscope (OLYMPUS BX53) was used to observe the red (*E. coli*) and green fluorescent bacteria (bacteria carrying the RP4 antibiotic resistance plasmid through horizontal gene transfer) extracted from mouse feces. The excitation wavelength for green fluorescence was 488 nm, with an emission wavelength of 500 nm - 550 nm. The excitation wavelength for red fluorescence was 561 nm, with an emission wavelength of 600 nm - 680 nm.

A 10 μL aliquot of the bacterial suspension, extracted and pre-treated from mouse feces, was aspirated using a micropipette. The bacterial suspension was evenly dropped onto the center of a glass slide, and a cover slip was placed at a 45-degree angle to avoid bubble formation. The fluorescent microscope was turned on, and the light source was adjusted to appropriate brightness. The glass slide was placed under the objective lens, and the sample's approximate location was first identified using a low magnification objective. Subsequently, the objective lens was switched to a higher magnification, and the distribution and morphology of green and red fluorescent bacteria were observed through fluorescence mode. The fluorescence microscope brightness and contrast were adjusted to ensure clear visibility of green and red fluorescence. Finally, images of green and red fluorescent bacteria were captured using the fluorescence microscope's built-in camera.

To accurately quantify conjugation frequency within a complex gut microbiome, bacterial fluorescence signals were detected using flow cytometry. The bacterial suspension was diluted to 5×10^6 CFU/mL with PBS and then analyzed on a flow cytometer (FACSAria™ Fusion, BD, USA) equipped with an argon ion laser. The PE-CF594 channel (610/20) was used to collect red fluorescence signals emitted by donor bacteria (*E. coli* MG1655, *mCherry*$^{+}$, *gfp*$^{-}$), while the FITC channel (530/30) collected green fluorescence signals emitted by transconjugants (*gfp*$^{+}$), which acquired the RP4 plasmid. To establish a reliable gating strategy, we first analyzed the fluorescence

profile of the donor strain (*E. coli* MG1655, *mCherry*$^{+}$, *gfp*$^{-}$), which only exhibited red fluorescence. This initial measurement defined the Q1 region, ensuring accurate differentiation of transconjugant (Q3, *gfp*$^{+}$) and recipient (Q4, no fluorescence) populations in subsequent analyses. The gating strategy of sorting the *gfp*-expressing cells from mating mixtures by a cell sorter (FACSAria™ Fusion, BD, USA). Using FlowJo_10.8.1 software for data analysis. The procedure consists of four consecutive gates with bivariate plots: Gate I was drawn to select only particles of bacterial sizes based on forward and side scatter (SSC-A vs FSC-A) (A); Gates II and III were drawn to remove any doublets and select singular bacterial cells using (SSC-W vs SSC-A) and (FSC-H vs FSC-A), respectively (B-C); Gate IV was analyzed using GFP-H vs PE-CF594-H (*mCherry*) fluorescence intensity, to distinguish between the donor (*mCherry*, Q1), recipient (no fluorescence, Q4), and transconjugant cells (*gfp*, Q3) (D). The plasmid conjugation frequency was calculated by using Eq. (1).

$$\text{conjugative transfer rate} = \frac{\text{conjugants (Q3, green fluorescent) count}}{\text{gut microbiome (Q4, non-fluorescent) bacterial count}} \quad (1)$$

Text S8 Detection of cytokines

The mouse intestinal contents were rinsed with PBS and then cut into small pieces. 50 μL of standard mixture at different concentrations was added to the standard well. 10 μL of intestinal content sample and 40 μL of sample diluent were added to the sample well. 100 μL of detection antibody was added to each sample. A sealing film was used to seal the reaction well, which was subsequently incubated at 37 °C for 60 minutes. After the reaction was complete, the solution was discarded, and 300 μL of washing solution was added to each well for 5 replications. 100 μL of the HRP enzyme was added to each sample, which was subsequently incubated at 37 °C for 30 minutes, after which the solution was discarded, and another 350 μL of washing solution was added for 5 replications. A total of 90 μL of TMB solution was added to each sample at 37 °C for 15 minutes. Finally, 50 μL of termination solution was added to each sample to terminate the reaction. The OD_{450} values were determined within 15 minutes. The concentrations of the four cytokines were calculated using external standard method. The standard curve equation for the four cytokines were listed in Table S8.

Table S8 The standard curve equations established in this study for the quantification of cytokines

Cytokines	Standard curve equations	R^2
IL-8	y = 4637.3x - 1132.2	0.98
IL-6	y = 579.11x - 98.281	0.97
1L-1β	y = 282.27x - 51.934	0.98
TNF-α	y = 282.42x - 47.525	0.97

Text S9 Detection of enrofloxacin

The mouse fecal samples collected from the same day were mixed, freeze-dried, ground and sieved with 150 μm screen. The 0.5 g fecal sample was weighed in a 50 mL centrifuge tube, added with 2.5 mL Na_2EDTA-Mcllvaine buffer (pH=4), and then added with 10 mL acetonitrile (1% formic acid) solution, vortexed for 2 min, ultrasonically extracted at 25 °C for 15 min. And then 1.5 g NaCl and 2.5 g anhydrous Na_2SO_4 were added into the centrifuge tube, vortexed for 2 min, and centrifuged at 4 °C for 8000 r/min to obtain the upper organic phase.

The upper organic phase (2 mL) was centrifuged into a 15 mL centrifuge tube, purified by adding 10 mg PSA, 10 mg EMR-Lipid and 5 mg CarbonS, vortexed for 2 min, and centrifuged at 8000 r/min for 5 min. 1 mL of the supernatant was passed through a 0.22 μm organic filter membrane, and the filtrate was analyzed on the machine. The sample pretreatment in the SHIME system was the same as above, which no ultrasonic process is required.

The HPLC-MS/MS condition were as follows: chromatographic column: Waters ACQUITY UPLC BEH C18 (100 mm×2.1 mm, 1.7 μm)； column temperature: 40 °C; flow rate: 0.3 mL/min; injection volume: 10 μL; mobile phase: formic acid aqueous solution (containing 0.5‰ formic acid) (mobile phase A), and acetonitrile (mobile phase B); Gradient elution procedure: Perform isocratic elution with 60% 0.5‰ (v/v) formic acid solution and 40% acetonitrile.

MRM mode was selected to optimize the mass spectrometry parameters such as collision energy, declustering voltage and capillary voltage, as shown in Table S9.

Table S9 Mass spectrometry parameter information

Compound	Retention time/min	Ionization/mode	Precursorion (m/z)	Productions (m/z)	Collision energies/eV
Enrofloxacin	0.74	ESI^+	360.2	316.4/245.1/342.3	28/35/30

Text S10 Code for correlation network analysis

This code constructs a co-occurrence network based on microbial correlation data and saves

the processed edge and node information into CSV files. First, the microbial correlation matrix and annotation data are read. Then, the annotation data is deduplicated, and the cleaned information is saved. Next, the Spearman correlation coefficients between microbes are calculated, and significant correlations (r > 0.60 and $P < 0.05$) are filtered. The next step involves adding additional information to the filtered edge data, such as the edge type and sign (positive or negative correlation), and saving this data to a CSV file. An undirected network graph is constructed, and node information is extracted. The genus name of each node is mapped to the corresponding phylum information, and node data is recorded. Ultimately, the code generates and saves network edge data (edges.csv) and node data (nodes.csv), providing a foundation for subsequent network analysis or visualization. The detailed code is:

```
library(linkET)
library(igraph)
library(dplyr)
setwd("C:/G")
network = read.table("SHIME(Mice).txt", head=T, row.names=1)
Bacteria <- readxl::read_xlsx("Annotation.xlsx", sheet = "Bacteria")
tax_mixed_0 <- dplyr::bind_rows(Bacteria)
id <- duplicated(tax_mixed_0$Genus)
tax_mixed <- tax_mixed_0 [!id, ]
write.table(tax_mixed,"tax_mixed.csv",row.names=FALSE,col.names=TRUE,sep=",")
edges0 <- network %>%
  correlate(method = "spearman") %>%
  as_md_tbl(type = "upper", diag = FALSE) %>%
  dplyr::filter(abs(r) > 0.6, p < 0.05)
edges <- edges0 %>%
  dplyr::mutate(Type = "Undirected",
              ID = seq_len(dplyr::n()),
              Label = "",
              sign = ifelse(r > 0, "P", "N"),
              abs_r = abs(r)) %>%
  dplyr::rename(Source = .rownames,
              Target = .colnames) %>%
  dplyr::select(Source, Target, Type, ID, Label, r, p, sign, abs_r)
write.table(edges,"edges.csv",row.names=FALSE,col.names=TRUE,sep=",")
graph <- graph_from_data_frame(d = edges, directed = FALSE)
nodes <- igraph::as_data_frame(graph, what = "vertices")
tax_map <- rlang::set_names(tax_mixed$Phylum, tax_mixed$Genus)
```

```
nodes$Phylum <- tax_map[nodes$name]
nodes %>%
  dplyr::rename(ID = name) %>%
  dplyr::mutate(Label = ID) %>%
  dplyr::filter(!is.na(Phylum)) %>%
  write.csv(file = "nodes.csv", row.names = FALSE)
```

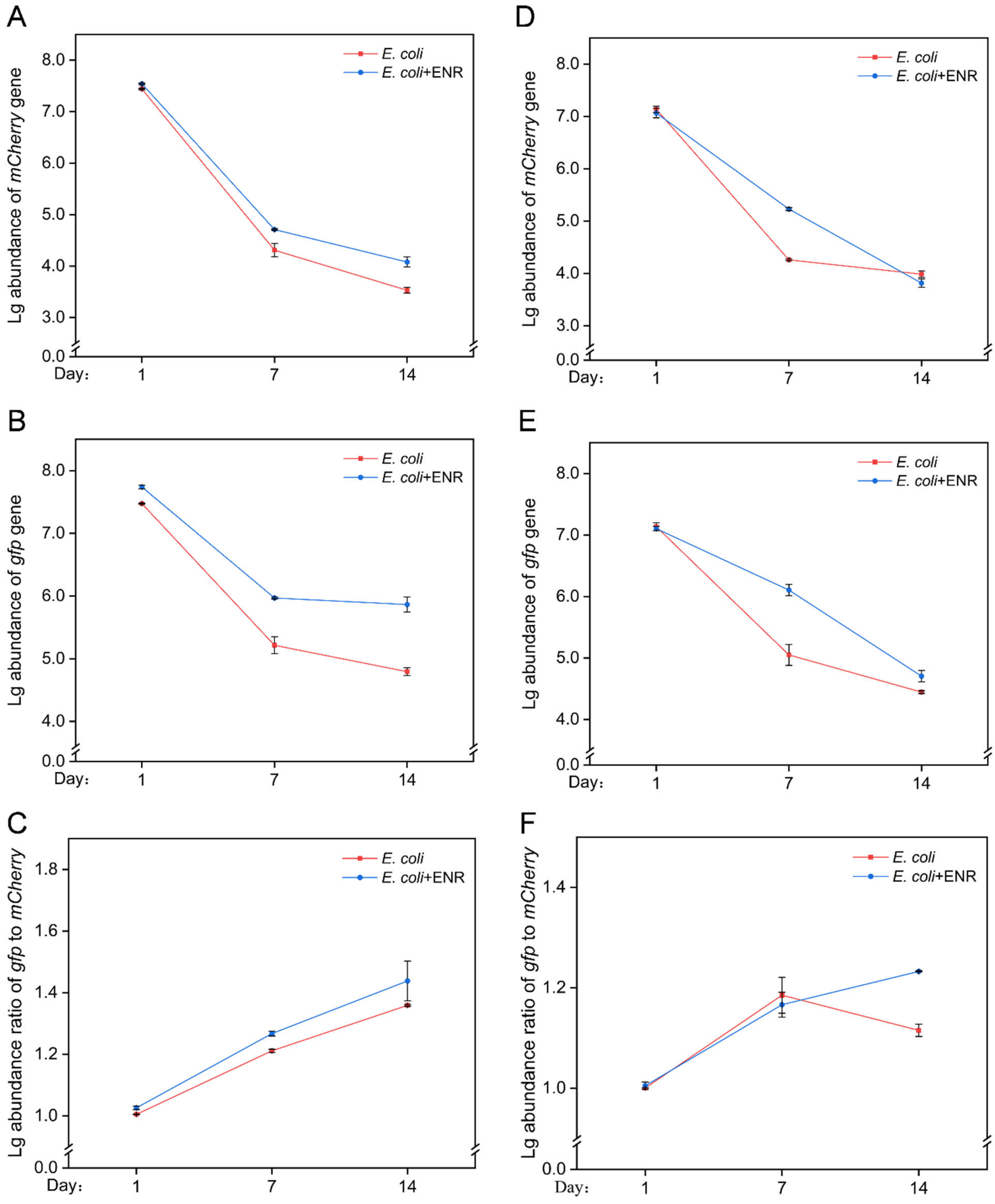

Figure S1 The abundance of *gfp* and *mCherry* (log10 transfer) in the SHIME system of transverse colon and descending colon. Logarithmic abundances of *mCherry* (**A**) and *gfp* (**B**), and the logarithmic abundance ratio of *gfp* gene to *mCherry* gene (**C**) in the transverse colon of the SHIME

system. Logarithmic abundances of *mCherry* (**D**) and *gfp* (**E**), and the logarithmic abundance ratio of *gfp* gene to *mCherry* gene (**F**) in the descending colon of the SHIME system.

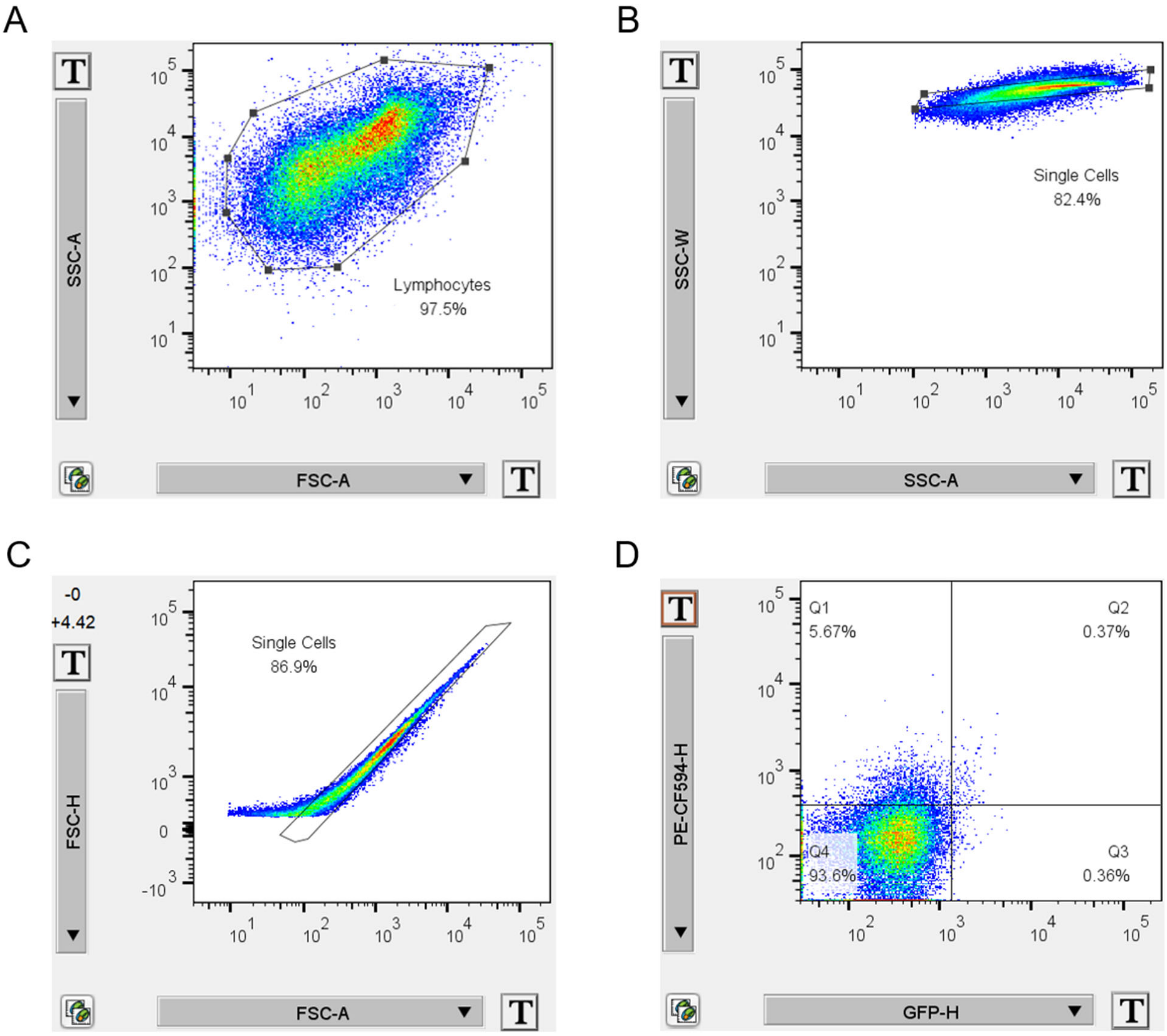

Figure S2 The gating strategy of sorting the *gfp*-expressing cells from mating mixtures by a cell sorter (FACSAria™ Fusion, BD, USA). UsingFlowJo_10.8.1 software for data analysis. The procedure consists of four consecutive gates with bivariate plots: Gate I was drawn to select only particles of bacterial sizes based on forward and side scatter (SSC-A vs FSC-A) (**A**); Gates II and III were drawn to remove any doublets and select singular bacterial cells using (SSC-W vs SSC-A) and (FSC-H vs FSC-A), respectively (**B-C**); Gate IV was analyzed using GFP-H vsPE-CF594-H (*mCherry*) fluorescence intensity, to distinguish between the donor (*mCherry*, Q1), recipient (no fluorescence, Q4), and transconjugant cells (*gfp*, Q3) (**D**).

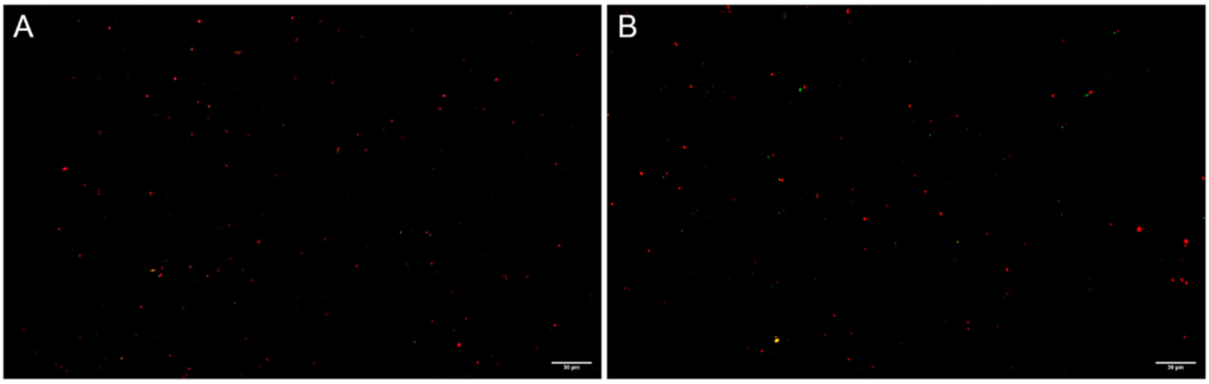

Figure S3 Fluorescence microscopy images of fecal bacterial *in vivo* mice experiments. The red-fluorescent bacteria were the donor *E. coli* labelled with a *mCherry* gene and the green fluorescent bacteria were transconjugants with newly acquired *gfp*-tagged RP4 plasmid. *E. coli* group (**A**) and *E. coli*+ENR group (**B**).

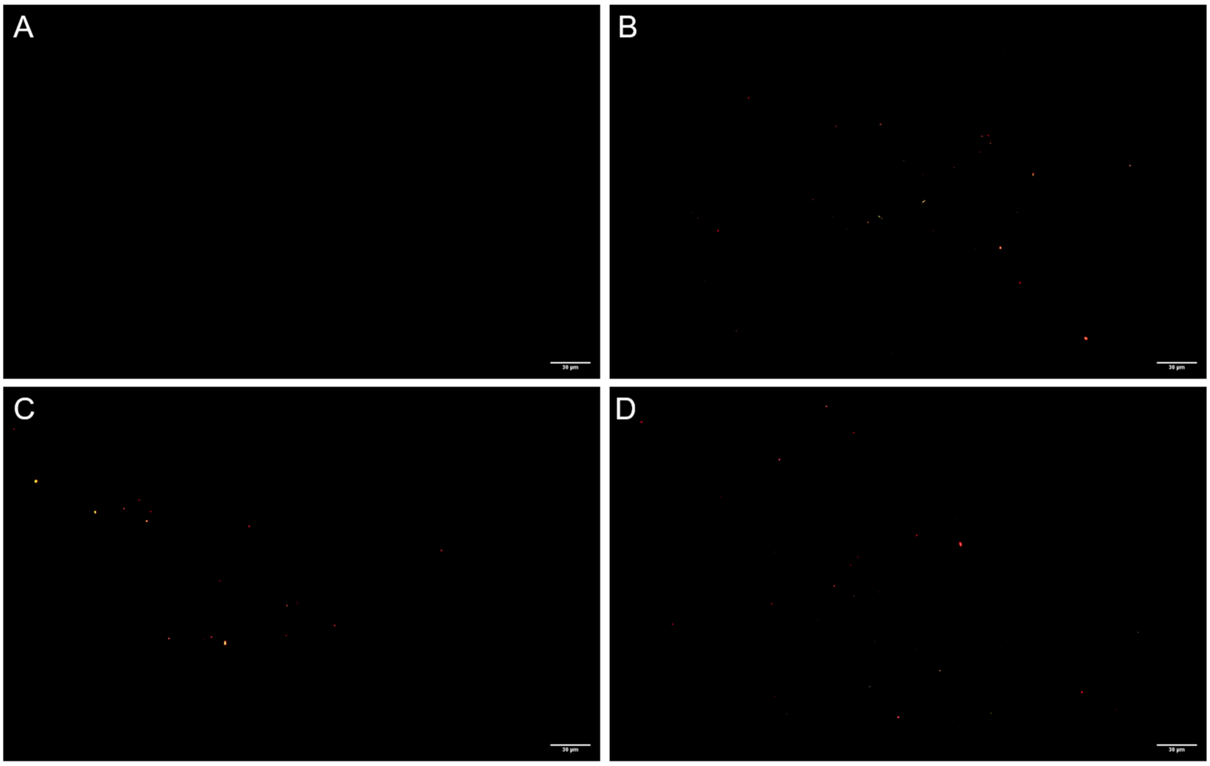

Figure S4 Fluorescence microscopy presents the dynamic process of red-fluorescent bacteria (the donor *E. coli* labelled with a *mCherry* gene) and green-fluorescent bacteria (transconjugants with

newly acquired *gfp*-tagged RP4 plasmid) in the intestinal contents of mice at different time points of the first gavage in *E. coli* group. 0 h (**A**), 2 h (**B**), 4 h (**C**) and 6 h (**D**).

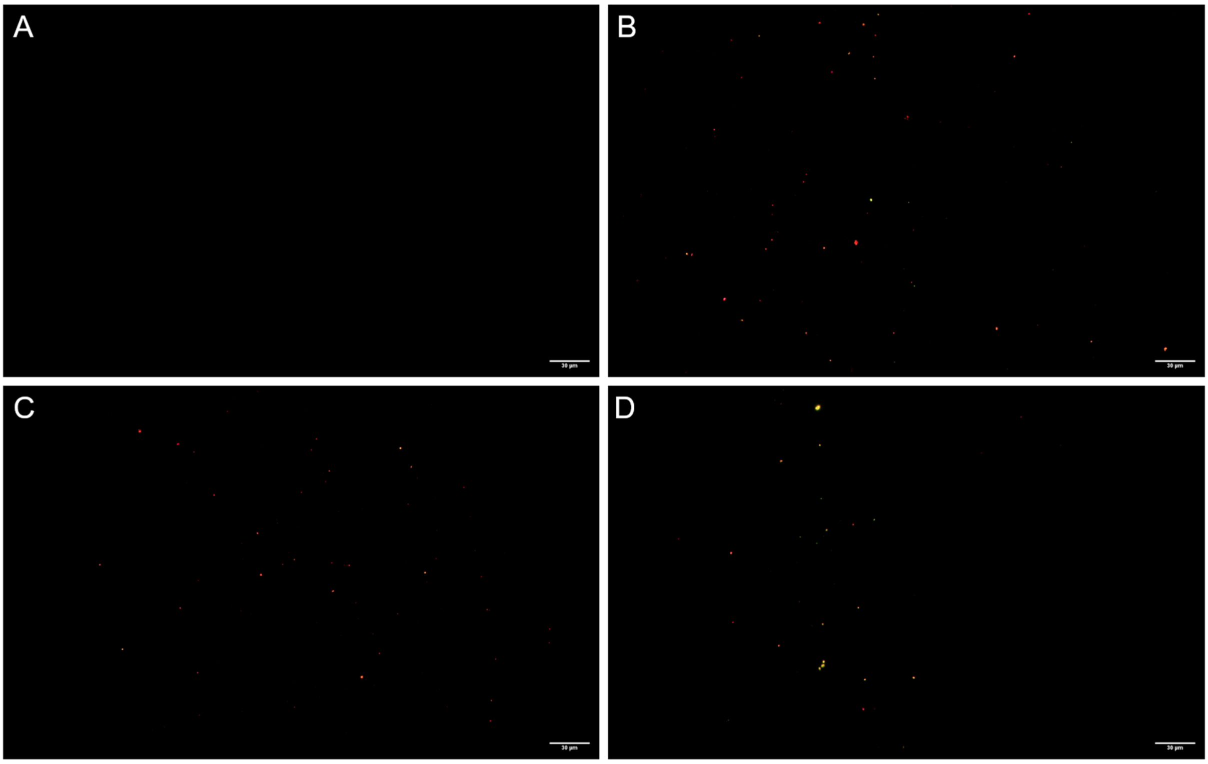

Figure S5 Fluorescence microscopy presents the dynamic process of red-fluorescent bacteria (the donor *E. coli* labelled with a *mCherry* gene) and green-fluorescent bacteria (transconjugants with newly acquired *gfp*-tagged RP4 plasmid) in the intestinal contents of mice at different time points of the first gavage in *E. coli*+ENR group mice. 0 h (**A**), 2 h (**B**), 4 h (**C**) and 6 h (**D**).

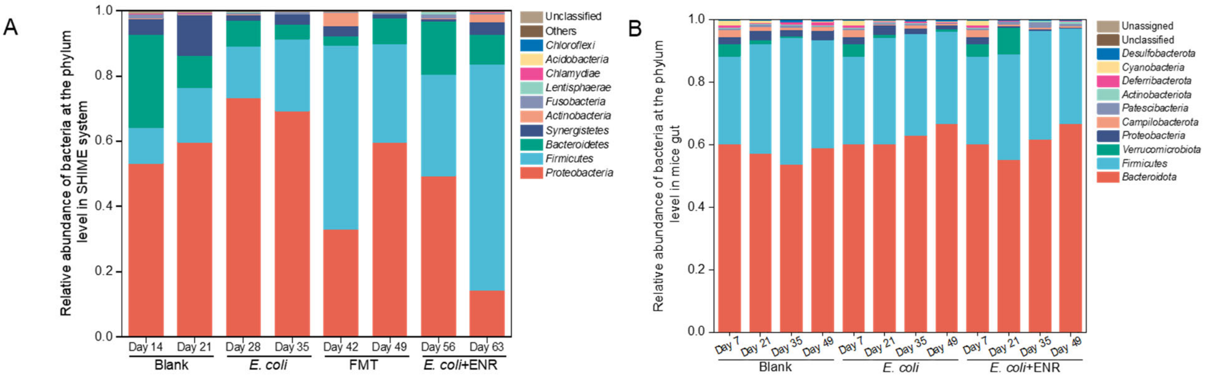

Figure S6 The bacteria composition detected in this study. A-B Community composition of gut bacteria at the phylum level. Ascending colon of the SHIME system (**A**) and mouse gut (**B**).

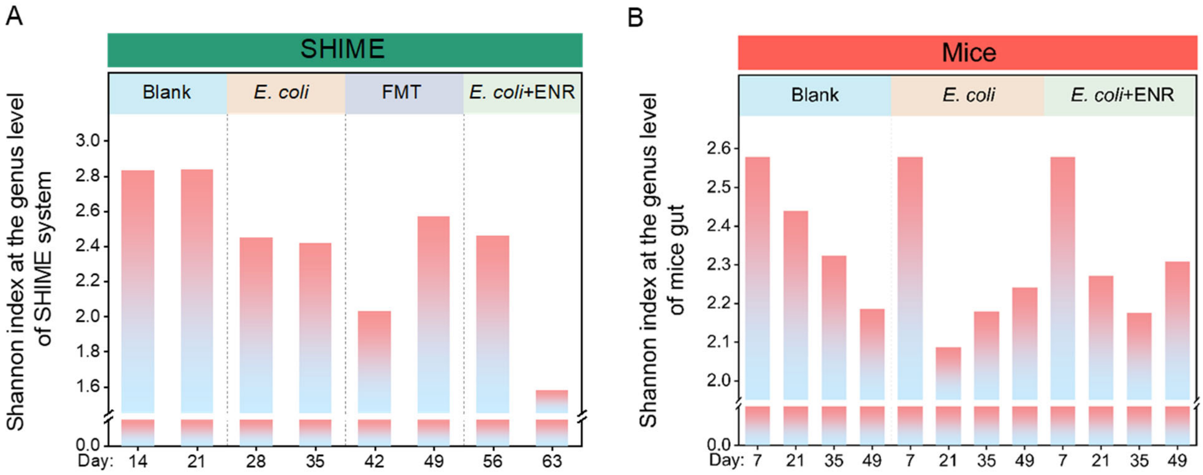

Figure S7 A-B Alpha diversity analysis of gut microbiota was conducted using the Shannon index to measure species richness and diversity. Ascending colon of the SHIME system (**A**) and mouse gut (**B**).

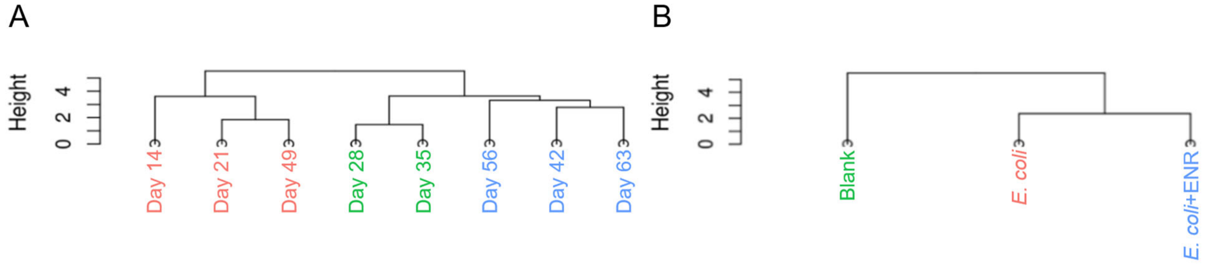

Figure S8 Hierarchical clustering analysis of ARGs detected in the SHIME system (**A**) and mouse gut (**B**).

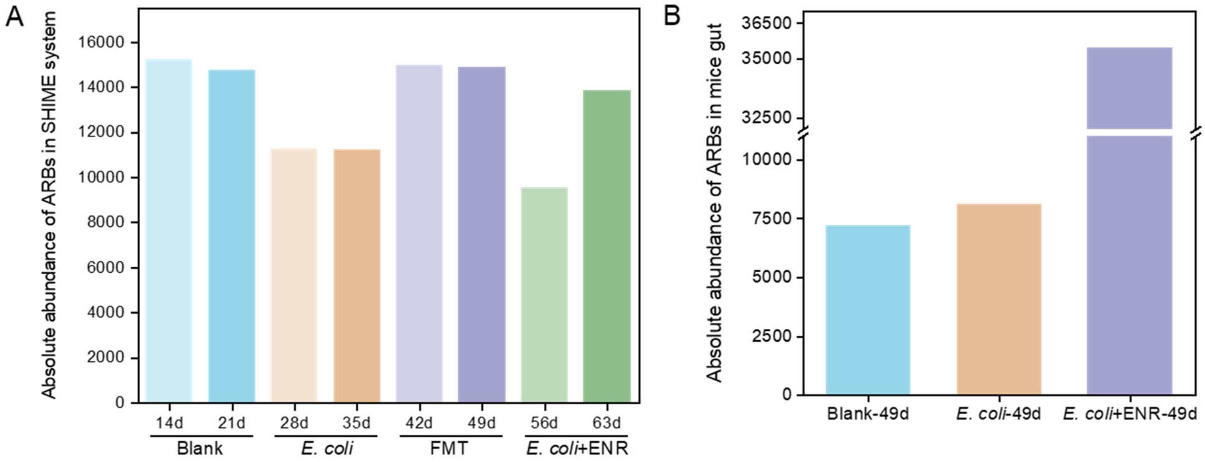

Figure S9 The absolute abundance of potential hosts of ARGs detected in the SHIME system (**A**) and mice experiments (**B**).

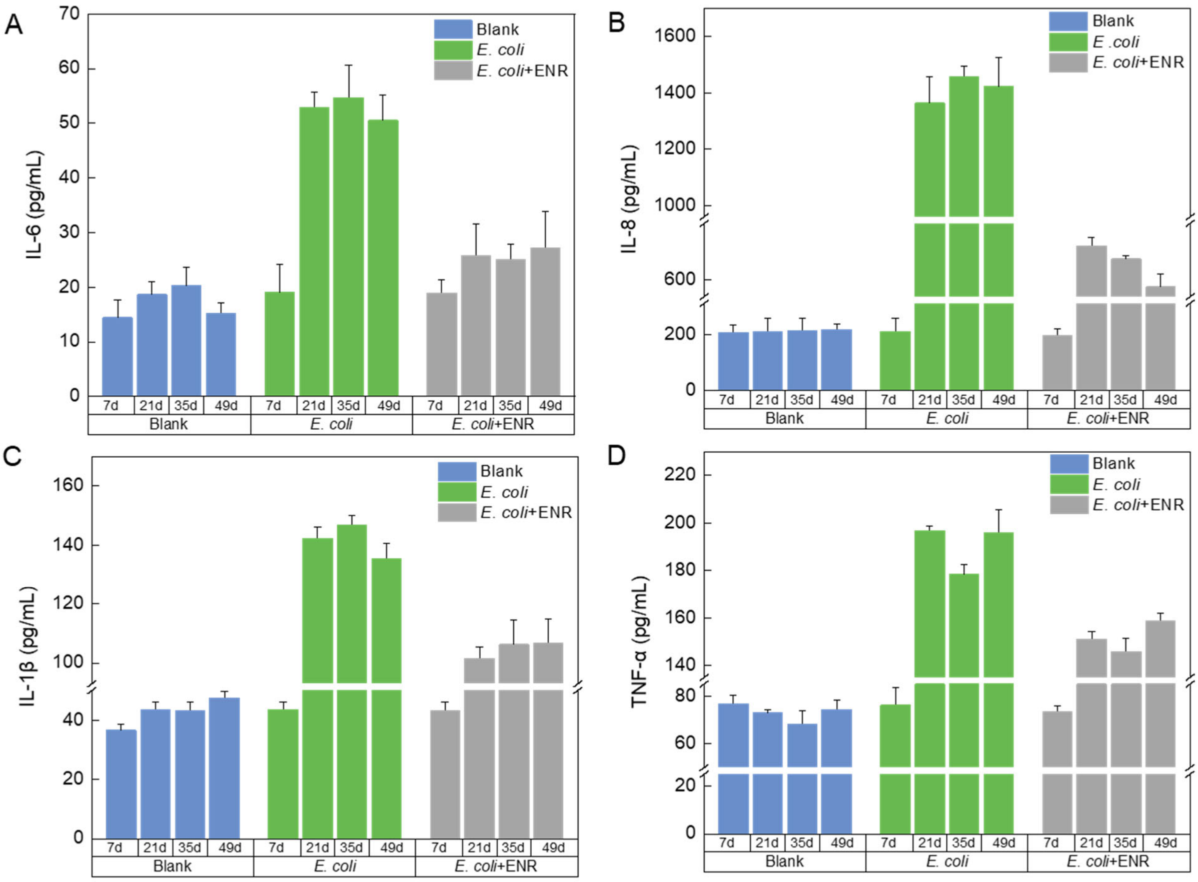

Figure S10 The concentration of four proinflammatory factors in the gut contents of mice. Chronic low-dose enrofloxacin exposure reduces the expression of pro-inflammatory cytokines IL-6, IL-8, IL-1β, and TNF-α in the gut microbiota. IL-6 (**A**), IL-8 (**B**), IL-1β (**C**), and TNF-α (**D**).

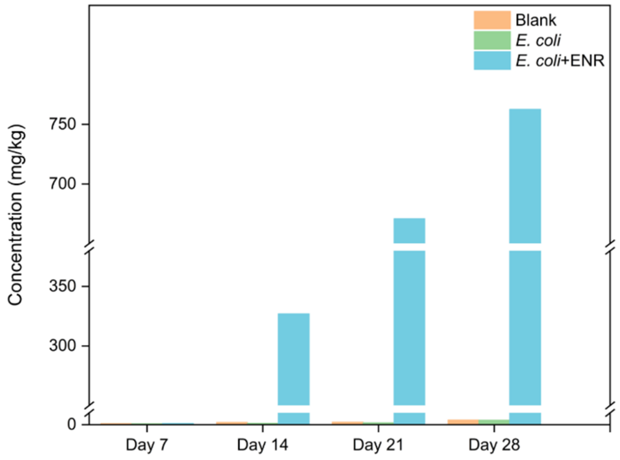

Figure S11 The concentration of enrofloxacin detected in the mice feces

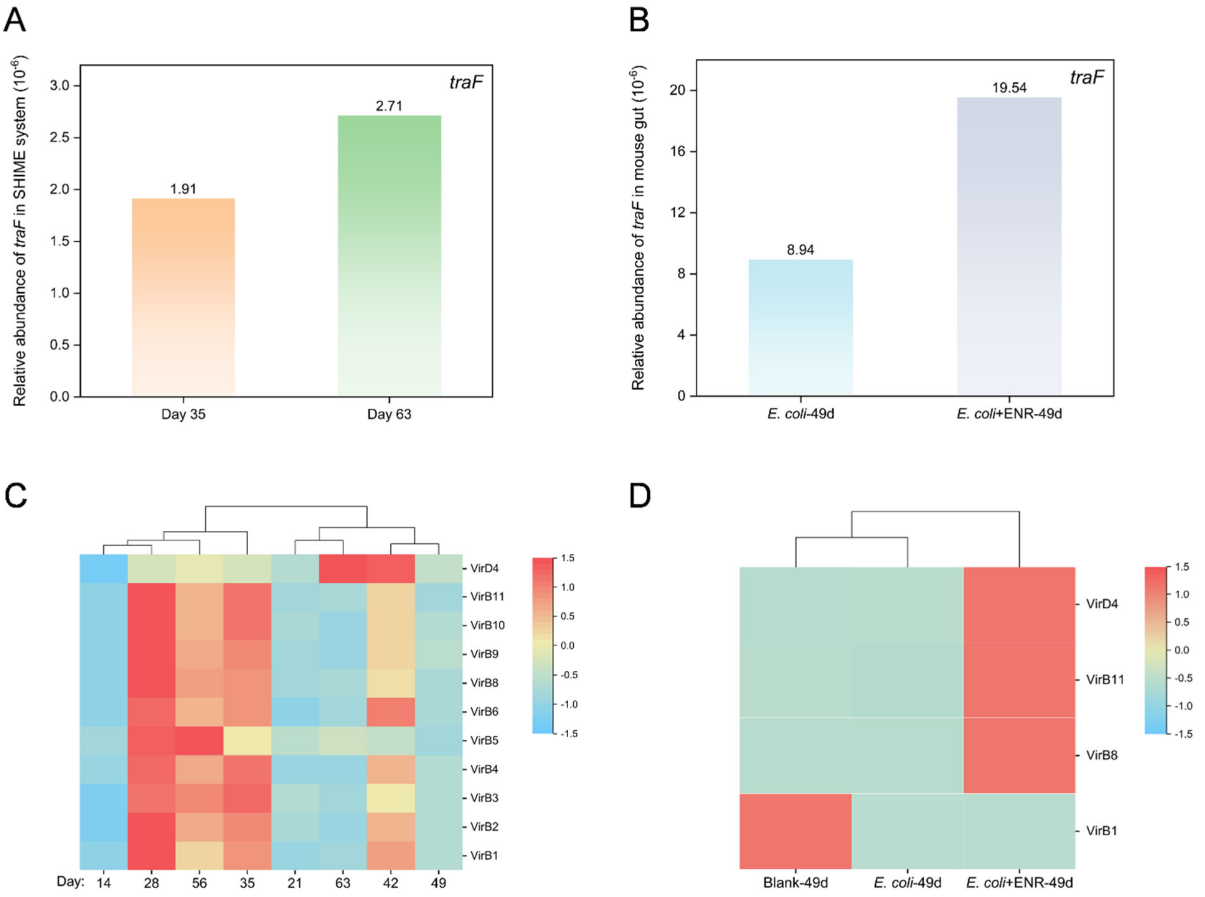

Figure S12 The abundance of *traF* detected *in vitro* SHIME experiments (**A**) and *in vivo* mice experiments (**B**); Heatmap analysis of the abundance of type IV secretion system-related genes *in vitro* SHIME experiments (**C**) and *in vivo* mice experiments (**D**).

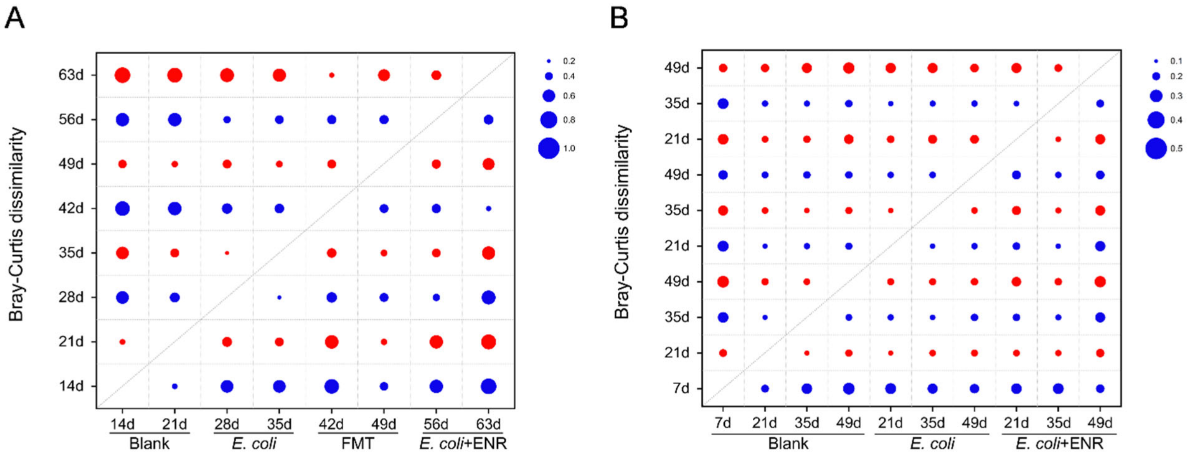

Figure S13 Beta diversity of gut microbiota at the genus level for all samples was assessed based on Bray-Curtis dissimilarity. In the bubble plot, the size of the circles represents the magnitude of the Bray-Curtis dissimilarity value; larger circles indicate greater beta diversity differences between samples. Ascending colon of the SHIME system (**A**) and mouse gut (**B**).
